# The pluripotent factor OCT4A enhances the self-renewal of human dental pulp stem cells by targeting lncRNA FTX in an LPS-induced inflammatory microenvironment

**DOI:** 10.1186/s13287-023-03313-8

**Published:** 2023-04-27

**Authors:** Hong Hong, Kai Zeng, Can Zhou, Xiaochuan Chen, Zhezhen Xu, Mengjie Li, Lu Liu, Qian Zeng, Qian Tao, Xi Wei

**Affiliations:** Hospital of Stomatology, Guanghua School of Stomatology, Guangdong Provincial Key Laboratory of Stomatology, Sun Yat-Sen University, Guangzhou, 510055 People’s Republic of China

**Keywords:** Dental pulp stem cells, OCT4A, LncRNA, FTX, Pulpitis

## Abstract

**Background:**

Regulating the pluripotency of human dental pulp stem cells (hDPSCs) is key for the self-repair of injured dental pulp. We previously found that OCT4A promotes the proliferation and odontogenic differentiation of human dental pulp cells (hDPCs). Recent studies have shown the interaction between OCT4A and lncRNAs in pluripotency maintenance of various stem cells. The aim of this study was to explore the underlying roles and mechanisms of OCT4A and its related lncRNAs in the proliferation and multidirectional differentiation of hDPSCs in an inflammatory microenvironment.

**Methods:**

Human lncRNA microarrays were applied to screen out the differentially expressed lncRNAs in hDPSCs between the OCT4A-overexpressing and vector groups. Lipopolysaccharide (LPS) was used to simulate the inflammatory microenvironment. The effects of OCT4A and the lncRNA FTX on the proliferation and multidifferentiation of hDPSCs were observed by the CCK-8 assay, EdU staining, real-time PCR, western blotting, and Alizarin red and oil red O staining. Bioinformatics analysis and chromatin immunoprecipitation (ChIP) assays were performed to clarify the targeted mechanism of OCT4A on FTX. The regulation by FTX of the expression of OCT4A and its downstream pluripotent transcription factors SOX2 and c-MYC was further detected by real-time PCR and western blotting.

**Results:**

The microarray results showed that 978 lncRNAs (250 of which were upregulated and 728 downregulated) were potentially differentially expressed genes (fold change ≥ 2, *P* < 0.05). LPS stimulation attenuated the self-renewal of hDPSCs. OCT4A enhanced the cell proliferation and multidifferentiation capacities of hDPSCs in an inflammatory microenvironment, while FTX exhibited the opposite effects. OCT4A negatively regulated FTX function by binding to specific regions on the FTX promoter, thereby inhibiting the transcription of FTX. Moreover, overexpression of FTX downregulated the expression of OCT4A, SOX2 and c-MYC, whereas knockdown of FTX facilitated their expression.

**Conclusions:**

OCT4A was found to be a crucial factor maintaining the self-renewal of hDPSCs by transcriptionally targeting FTX in an inflammatory microenvironment. Moreover, we proposed a novel function of FTX in negatively regulating the pluripotency and multilineage differentiation capacity of hDPSCs. The hierarchical organization between OCT4A and FTX expanded the understanding of the network between transcription factors and lncRNAs in fine-tuning the pluripotency/differentiation balance of adult stem cells, and provided prospective targets for optimizing dental-derived stem cell sources for regenerative endodontics.

**Supplementary Information:**

The online version contains supplementary material available at 10.1186/s13287-023-03313-8.

## Background

Dental pulp inflammation, also known as pulpitis, is the most common inflammatory disease that leads to the destruction of pulp tissue and loss of tooth function. Human dental pulp stem cells (hDPSCs) are a type of adult stem cell existing in dental pulp tissues with an immunophenotype and multidirectional differentiation characteristics similar to those of bone marrow mesenchymal stem cells (BMSCs) [1]. When the dental pulp microenvironment is invaded by bacteria or undergoes trauma, DPSCs are prone to migrating toward the injured site and differentiating into odontoblasts, secreting reparative dentin to protect the dental pulp [2, 3]. Several clinical and preclinical animal studies have also demonstrated important prospects for pulp regeneration using DPSCs in pulpitis [4–6]. Therefore, it would be of great significance to identify targets for optimizing the stemness property of hDPSCs for the repair and regeneration of injured dental pulp.

The differentiation of stem cells is regulated by a network of nuclear transcription factors, epigenetic factors and signaling pathways. Octamer-binding transcription factor 4A (OCT4A), the original form of OCT4 which belongs to class V of the POU (PIT/OCT/UNC) transcription factor family, has been proven to be the key transcription factor for the maintenance of self-renewal and pluripotency of embryonic stem cells (ESCs) and adult stem cells, and is closely relating to the processes of cell differentiation and injury repair [7]. Our previous study showed that OCT4A played a vital role in the promotion of pluripotency and multidirectional differentiation capability of human dental pulp cells (hDPCs) [8]. However, the specific molecular mechanism requires further exploration.

Long noncoding RNAs (lncRNAs) are a class of noncoding RNAs that are more than 200 bp in length, and are implicated in the complex molecular circuitry in diverse biological processes [9, 10]. A growing body of evidence has demonstrated that lncRNAs act as an essential part of the pluripotency controlling network and specific lineage commitment of ESCs or adult stem cells [11, 12]. A recent study showed that lncRNA Gm15055 was obviously induced by OCT4 and interacted with PRC2 to maintain H3K27me3 levels on HoxA genes to modulate embryonic pattern formation of mouse ESCs [13]. LncRNA NEAT1 impaired OCT4/SOX2 complex stability to dysregulate downstream transcription networks of pluripotency maintenance in aged BMSCs [14]. The OCT4 pseudogene lncRNA Oct4P4 can form a complex with the SUV39H1 HMTase to silence the OCT4 gene and reduce mouse ESC self-renewal [15]. These studies indicated that the interaction between OCT4 and lncRNAs may play an important role in cell pluripotency maintenance.

Accordingly, we hypothesized that lncRNAs were also involved in the molecular network of OCT4A to regulate the repair of injured dental pulp. In the present study, we utilized high-throughput microarrays to identify potential lncRNAs related to OCT4A and investigated functions of both OCT4A and candidate lncRNAs in the proliferation and multidirectional differentiation of hDPSCs under inflammatory conditions. Furthermore, we explored how OCT4A interacts with the candidate lncRNAs to reveal their potential regulatory mechanisms on the pluripotency of hDPSCs.

## Methods

### Cell culture

hDPSCs were cultured as described previously [16]. Healthy human premolars and impact third molars were collected from 18- to 25-year-old patients who were experiencing tooth extractions for the purpose of orthodontic treatment at the Hospital of Stomatology, Sun Yat-sen University. Informed consent was obtained from all donors. All experimental protocols were performed according to the guidelines of the Ethics Review Board. hDPSCs were cultured in Dulbecco’s modified Eagle’s medium (DMEM; Gibco, USA) with 20% fetal bovine serum (FBS; Gibco, Australia), and incubated at 37 °C in 5% CO_2_. Cells from passages 3 to 5 were used in the following experiments.

### Flow cytometric analysis of surface markers

Flow cytometric analysis was conducted to identify the phenotype of hDPSCs. The MSC phenotyping cocktail is comprised of both positive (CD29-FITC, CD44-PE, and CD90-PE-CY5, BD Bioscience, USA) and negative (CD34-PE and CD45-PE-CY5, BD Bioscience, USA) fluorochrome-conjugated monoclonal antibodies. IgG1-FITC, IgG1-PE-CY5 and IgG1-PE (BD Bioscience, USA) were used as isotype controls. hDPSCs were suspended to 4 × 10^5^ cells/mL, incubated with different antibodies for 30 min at 4 °C, resuspended in FACS buffer, and analyzed using a MOFloTM high-performance cell sorter (Beckman Coulter, USA).

### Establishment of OCT4A+ /hDPSCs or OCT4A-/hDPSCs, and FTX+ /hDPSCs or FTX-/hDPSCs

For OCT4A overexpression (OCT4A+ /hDPSCs), the coding domain sequence region of the OCT4A gene was amplified and cloned into a replication-deficient lentivector (GenePharma, China) carrying a green fluorescent protein (GFP) reporter gene. For OCT4A knockdown (OCT4A-/hDPSCs), 3 different OCT4A shRNA sequences (Table 1) named shRNA1, shRNA2 and shRNA3 were designed to construct recombinant shuttle plasmid and cloned into a replication-deficient lentivector (GenePharma, China) to obtain the stable knockdown effect of OCT4A in hDPSCs.

**Table 1:** Three different shRNA sequences of the *OCT4A* gene

shRNA	Sequence
*shRNA1*	
Sense	GATCCGAAGGATGTGGTTCGAGTATTCAAGAGATACTCGAACCACATCCTTCTTTTTTG
Antisense	AATTCAAAAAAGAAGGATGTGGTTCGAGTATCTCTTGAATACTCGAACCACATCCTTCG
*shRNA2*	
Sense	GATCCGCGGACCTGGCTAAGCTTCCAATTCAAGAGATTGGAAGCTTAGCCAGGTCCGCTTTTTTG
Antisense	AATTCAAAAAAGCGGACCTGGCTAAGCTTCCAATCTCTTGAATTGGAAGCTTAGCCAGGTCCGCG
*shRNA3*	
Sense	GATCCGTGGCTTCGGATTTCGCCTTCTTTCAAGAGAAGAAGGCGAAATCCGAAGCCACTTTTTTG
Antisense	AATTCAAAAAAGTGGCTTCGGATTTCGCCTTCTTCTCTTGAAAGAAGGCGAAATCCGAAGCCACG

For FTX overexpression (FTX+ /hDPSCs), the coding domain sequence region of the FTX gene was amplified and cloned into the pHBLV-CMVIE-Puro lentivector (Hanbio, China) to transfect hDPSCs. RNA interference (RNAi) was performed for knockdown of FTX (FTX-/hDPSCs) by using a commercial RiboTM human-FTX Smart Silencer (Ribo, China), which contained 6 different FTX RNAi target sequences (Table 2). hDPSCs were transfected with human-FTX Smart Silencer by using Lipofectamine RNAiMAX (Invitrogen, Life Technologies, USA).

**Table 2:** Six FTX interference target sequences for RNAi

Silencer	Sequence
Silencer 1	GATCTGCCTGTTACTCATA
Silencer 2	TGTCCACGTATCACAGAGG
Silencer 3	CTTCAGGTGTAACATAAGG
Silencer 4	ATTCCTGCTACGACACTGAA
Silencer 5	TTAAAACATCCTTGCCTCAG
Silencer 6	GATTGTGCCGCTGTTAGACG

The expression of GFP in hDPSCs was visualized using a fluorescence microscope (Zeiss, Germany) after cell transfection for 48 h. After being cultured with 1 μg/mL puromycin (Hanbio, China) for one week, the expression of OCT4A or FTX was detected in OCT4A overexpressing, OCT4A knockdown or FTX overexpressing cells. FTX levels in FTX knockdown hDPSCs were detected by real-time PCR after transfection with human-FTX Smart Silencer for 24, 48 and 72 h.

### LncRNA microarrays

Total RNA was isolated from OCT4A-overexpressing and vector hDPSCs. Human lncRNA microarrays (KangChen Bio-tech, China) were used to screen the differentially expressed lncRNAs in hDPSCs between the OCT4A- overexpressing and vector groups. The array image analysis and subsequent data processing were performed by Agilent Feature Extraction software (version 11.0.1.1) and the GeneSpring GX v11.5.1 software package (Agilent Technologies, USA). Differentially expressed lncRNAs and mRNAs with statistically significant differences (fold change > 2.0 or < − 2.0, *P* < 0.05) between the two groups were identified and distinguished by volcano plot filtering and hierarchical clustering. Gene Ontology (GO) and Kyoto Encyclopedia of Genes and Genomes (KEGG) pathway analyses were applied to investigate the roles of the differentially expressed genes in GO terms or biological pathways.

Five differentially expressed lncRNAs, namely, downregulated FTX, SNHG6 and SUZ12P1 and upregulated MT1DP and ST3GAL3, were selected for further validation by real-time PCR, and related differentially expressed mRNAs were selected to construct coding-noncoding gene coexpression (CNC) networks using Cytoscape 2.8.3 software (The Cytoscape Consortium, USA). The lncRNA-mRNA coexpression networks were identified by Pearson correlation coefficient values | r |≥ 0.9 and *P* < 0.05.

### Fluorescence in situ hybridization

For the detection of subcellular localization of lncRNA FTX, hDPSCs were incubated on slides and hybridized with the probe overnight. 4′,6-Diamidino-2-phenylindole (DAPI) solution (KeyGEN Biotech, China) was used for cell nuclei staining. The cells were imaged under a fluorescence inversion microscope (Zeiss, Germany).

### Chromatin immunoprecipitation (ChIP) assay

To explore the potential mechanism of OCT4A on FTX expression, the online databases JASPAR (http://jaspar.genereg.net) and PROMO (http://alggen.lsi.upc.es/cgibin/promo_v3/promo/promoinit.cgi?dirDB=TF_8.3) were used to predict the possible binding regions of OCT4A and the FTX promoters. A ChIP Kit (Axl-Biotech, China) was used to perform ChIP assays according to the manufacturer’s instructions. In brief, cells were incubated with 1% formaldehyde (Solarbio, China) for 10 min for DNA–protein cross-link generation. Cell lysates were then collected and sonicated to yield genomic DNA fragments of 200–600 bp and immunoprecipitated with an OCT4A antibody (Abcam, UK). Normal rabbit IgG was used as a control. qPCR was conducted using ChamQ SYBR qPCR Master Mix (Vazyme, China). Agarose gel (Biowest, France) electrophoresis was used to analyze the PCR products.

### Cell proliferation

A total of 1 μg/mL LPS derived from *E.coli* (Sigma-Aldrich, USA) was used to simulate the inflammatory microenvironment for the subsequent experiments. hDPSCs were stimulated with LPS for 1–7 days. Cell proliferation was measured using the Cell Counting Kit-8 (CCK-8) assay and 5-ethynyl-2ʹ-deoxyuridine (EdU) staining. For the CCK-8 assay, briefly, 3 × 10^3^ cells were cultured on 96-well plates and incubated with 100 µL of DMEM containing 10 µL of CCK-8 (Beyotime, China) solution for 2 h. Cell proliferation was detected by a quantitative assay at an absorbance of 450 nm. For EdU staining, DNA replication activity was evaluated by using an EdU detection kit (KeyGEN BioTECH, China) according to the manufacturer’s instructions. hDPSCs were seeded into a glass-bottom plate at a density of 1 × 10^5^ cells in 12-well plates and were treated with LPS for 24 h, followed by incubation with 500 μL of medium containing 50 μM EdU for 2 h at 37 °C. Then, the cells were fixed with 4% paraformaldehyde (Biosharp, China) for 30 min. After washing with PBS three times, the cells were permeabilized with 0.5% Triton X-100 (Solarbio, China) in PBS for 20 min. The nuclei were stained with Hoechst 33,342 dye (KeyGEN BioTECH, China). The cells were observed under a confocal fluorescence microscope (Olympus FV3000, Japan).

### Odontogenic differentiation

Once the cells reached 70% confluence, hDPSCs were induced in commercial osteogenic medium (Cyagen Biosciences, China) for 7, 14 and 21 days. The expression of the odontogenic-related markers alkaline phosphatase (ALP), osteocalcin (OCN), dentin sialophosphoprotein (DSPP) and dentin matrix protein-1 (DMP-1) was monitored by real-time PCR. Western blotting was performed to quantify DSPP and DMP-1. Cells were incubated in 1% Alizarin red solution (Cyagen Biosciences, China) for 5 min, and imaged with a stereomicroscope (Olympus, Japan) to detect mineralized nodule formation. The stain was desorbed with 10% cetylpyridinium chloride (Sigma‒Aldrich, USA) for 1 h and assessed by the absorbance at a 562-nm wavelength.

### Adipogenic differentiation

For adipogenesis, hDPSCs reaching 80% confluence were treated with commercial adipogenic induction medium (Cyagen Biosciences, China) for 3 days, followed by adipogenic maintenance medium (Cyagen Biosciences, China) for 1 day. After completing the three cycles of induction and maintenance, the cells were incubated in adipogenic maintenance medium for another 7 days. After 21 days of induction, the adiopogenic capacity was assessed by performing oil red O (Cyagen Biosciences, China) staining. The expression of the adipogenic-related markers peroxisome proliferator-activated receptor-gamma 2 (PPARγ-2) and lipoprotein lipase (LPL) were monitored by real-time PCR and western blotting.

### Chondrogenic induction

For chondrogenesis, hDPSCs reaching 80% confluence were treated with commercial chondrogenic induction medium (Cyagen Biosciences, China) for 21 days. Alcian blue staining (Cyagen Biosciences, China) was performed for the detection of chondrogenesis. The cell images were captured with a stereomicroscope (Olympus, Japan).

### Real-time PCR assay

Total RNA was isolated using RNAzol reagent (MRC, Inc., USA). cDNA was synthesized from 1 μg of total RNA using a reverse transcriptase master mix (TaKaRa, USA) in a total volume of 20 μL. Gene expression was measured by real-time PCR with SYBR Green (Roche, Germany). Primers were synthesized by the Beijing Genomics Institute (Shenzhen, China) and are listed in Table 3. Glyceraldehyde 3-phosphate dehydrogenase (GAPDH) was used as an internal control. The data were acquired and analyzed using a LightCycler 480 system.

**Table 3:** Primers used in the real-time fluorescent quantitative PCR

Gene	Primer sequence
*OCT4A*	Forward:5′- TGGGCCAGGCTCTGAGGTGT -3′
	Reverse:5′- TCCTGCTTCGCCCTCAGGCT -3′
*FTX*	Forward: 5′-CGGCGATTCTGGAGAGGTTG-3′
	Reverse: 5′-GGCATCACCTCCTGGTTGAA -3′
*SOX2*	Forward: 5′-GAGAACCCCAAGATGCACAAC-3′
	Reverse: 5′-CGCTTAGCCTCGTCGATGA-3′
*c-MYC*	Forward: 5′-GGCTCCTGGCAAAAGGTCA-3′
	Reverse: 5′-AGTTGTGCTGATGTGTGGAGA-3′
*SNHG6*	Forward: 5′-GTTAGTCATGCCGGTGTGGT -3′
	Reverse: 5′-AATACATGCCGCGTGATCCT -3′
*SUZ12P1*	Forward: 5′-TCCCACAGAGTGACTCGTCC-3′
	Reverse: 5′-TTCCAGAACATTGTGGGAGC-3′
*MT1DP*	Forward: 5′-TTCTGAGGCGAGAGGACTGA-3′
	Reverse: 5′-CAGCTGCACTTCTCCAATGC-3′
*ST3GAL3*	Forward: 5′-AAGCTGCACATTGACCATCATA-3′
	Reverse: 5′-GTGCGTCCAGGACACTCTC-3′
*ALP*	Forward:5′- GTTGACACCTGGAAGAGCTT -3′
	Reverse:5′- GTTCCTGTTCAGCTCGTACTG -3′
*OCN*	Forward:5′- AGCAAAGGTGCAGCCTTTGT -3′
	Reverse:5′- GCGCCTGGGTCTCTTCACT -3′
*DSPP*	Forward:5′- GGGATGTTGGCGATGCA -3′ Reverse:5′- CCAGCTACTTGAGGTCCATCTTC -3′
*DMP-1*	Forward:5′-ACATCAACCTGATTTTTGAGACTT-3′
	Reverse:5′-GGGTCTTCATTTGCCAAGGG-3′
*PPARγ2*	Forward:5′- GGCTTCATGACAAGGGAGTTTC -3′
	Reverse:5′- AACTCAAACTTGGGCTCCATAAAG -3′
*LPL*	Forward:5′- TGTGGTGGACTGGCTGTCA -3′
	Reverse:5′- CTGTCCCACCAGTTTGGTGTAG -3′
*GAPDH*	Forward:5′- CTGGGCTACACTGAGCACC -3′
	Reverse:5′- AAGTGGTCGTTGAGGGCAATG -3′

### Western blotting

Cells from each set of experiments were harvested and proteins were collected by lysis with phosphatase and protease inhibitors (Thermo Fisher Scientific, USA). Thirty micrograms of protein was separated by sodium dodecyl sulfate–polyacrylamide gel electrophoresis (GeneScript, USA) and transferred to polyvinylidene fluoride membranes (Millipore, USA). The membranes were incubated with specific antibodies overnight at 4 °C, and then were incubated with the horseradish peroxidase-conjugated secondary antibody (1:5000; Emarbio, China). Immunoreactive proteins were visualized by electrogenerated chemiluminescence detection agents (Millipore, USA). ImageJ version 1.50i software (Bethesda, USA) was used to quantify the densities of the protein bands. The following antibodies were used: OCT4A (1:1000; Abcam, UK), DSPP (1:1000; Santa Cruz, USA), DMP-1 (1:500; Abcam, UK), PPARγ-2 (1:500; Cell Signaling Technology, USA), LPL (1:500; Cell Signaling Technology, USA), SOX2 (1:1000; Abcam, UK), c-MYC (1:1000; Abcam, UK), Vinculin (1:5000; Cell Signaling Technology, USA), GAPDH (1:1000; ZENBIO, China) and β-actin (1:1000; Servicebio, China).

### Statistical analysis

All experiments were repeated at least three times. The data are shown as the means ± standard deviations (SDs) and were analyzed with SPSS 22.0 software (SPSS, USA). Statistical analyses were performed by analysis of variance, followed by Student’s *t* test for pairwise comparisons and one-way analysis of variance (ANOVA) for multiple comparisons. Statistical significance was defined as* P* < 0.05.

## Results

### Identification of hDPSCs

hDPSCs were successfully isolated from dental pulp tissues (Additional file 1: Fig. S1A). The cells exhibited spindle-shaped, fibroblast-like morphological properties, and there was no distinct change between passages 0 and passages 5 of hDPSCs (Additional file 1: Fig. S1A). Flow cytometric analysis showed high expression levels of the MSC markers CD29 (99.9%), CD44 (99.9%) and CD90 (100%) of hDPSCs, whereas negative expression was observed for the hematopoietic markers CD34 (0.4%) and CD45 (0.9%) (Additional file 1: Fig. S1B). In addition, after 21-days of osteogenic, adipogenic or chondrogenic induction, hDPSCs formed mineralized nodules that were detected by Alizarin red staining and lipid droplets as observed by oil red O staining, as well as proteoglycan accumulation in cartilage extracellular matrix visualized by Alcian blue staining (Additional file 1: Fig. S1C). These results demonstrated the stem cell characteristics and multidirectional differentiation capabilities of hDPSCs.

### OCT4A promoted the proliferation and multidirectional differentiation capabilities of hDPSCs under LPS stimulation

**Fig. 1:**
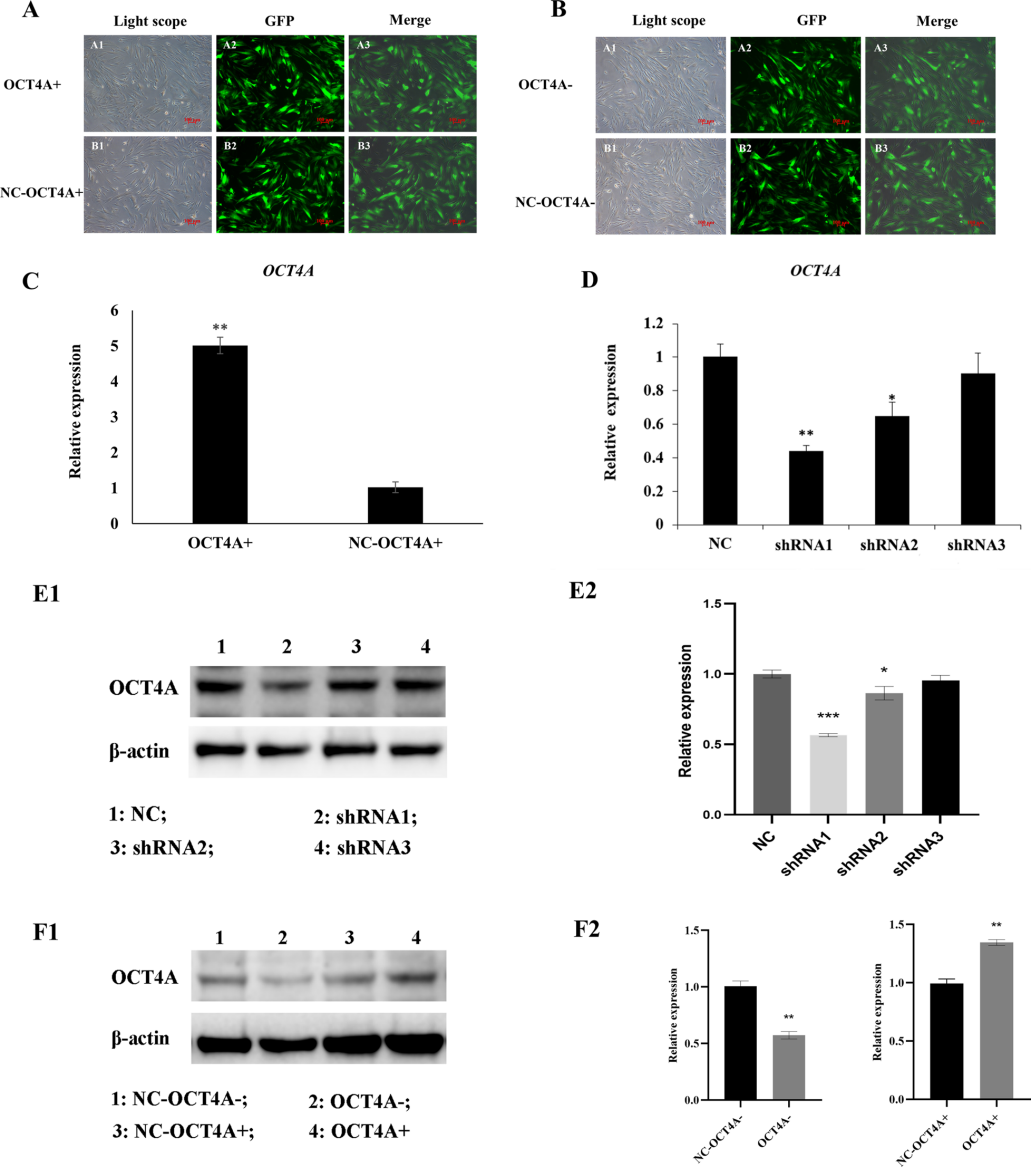
Establishment of OCT4A+ /hDPSCs or OCT4A- /hDPSCs. **A**, **B** Green fluorescence of the GFP plasmid in hDPSCs with OCT4A overexpression or knockdown (× 50). **C**, **D** Expression of the OCT4A gene in the OCT4A-overexpressing group or 3 different OCT4A shRNA groups. **E**, **F** Expression of OCT4A protein in the OCT4A-overexpressing group or 3 different OCT4A shRNA groups. Full-length blots are presented in Additional file 4. *: compared with the vector group, *P* < 0.05, **: compared with the vector group, *P* < 0.01; ***: compared with the vector group, *P* < 0.001

We next treated hDPSCs with lentivirus to strengthen or interfere with OCT4A expression. Strong GFP green fluorescence of transfected cells was detected under the fluorescence microscope in both the OCT4A-overexpressing and OCT4A-knockdown groups (Fig. 1A, B). Real-time PCR and western blot results revealed that the mRNA and protein expression levels of OCT4A in the OCT4A-overexpressing groups were significantly increased compared with those in the vector groups (Fig. 1C, F, **P < 0.01). The 50% interference efficiency of OCT4A in the shRNA1 group was shown by real-time PCR and western blotting (Fig. 1D, E, **P < 0.01). Therefore, the lentivirus vector shRNA1 was chosen to construct OCT4A knockdown hDPSCs for subsequent experiments.

**Fig. 2:**
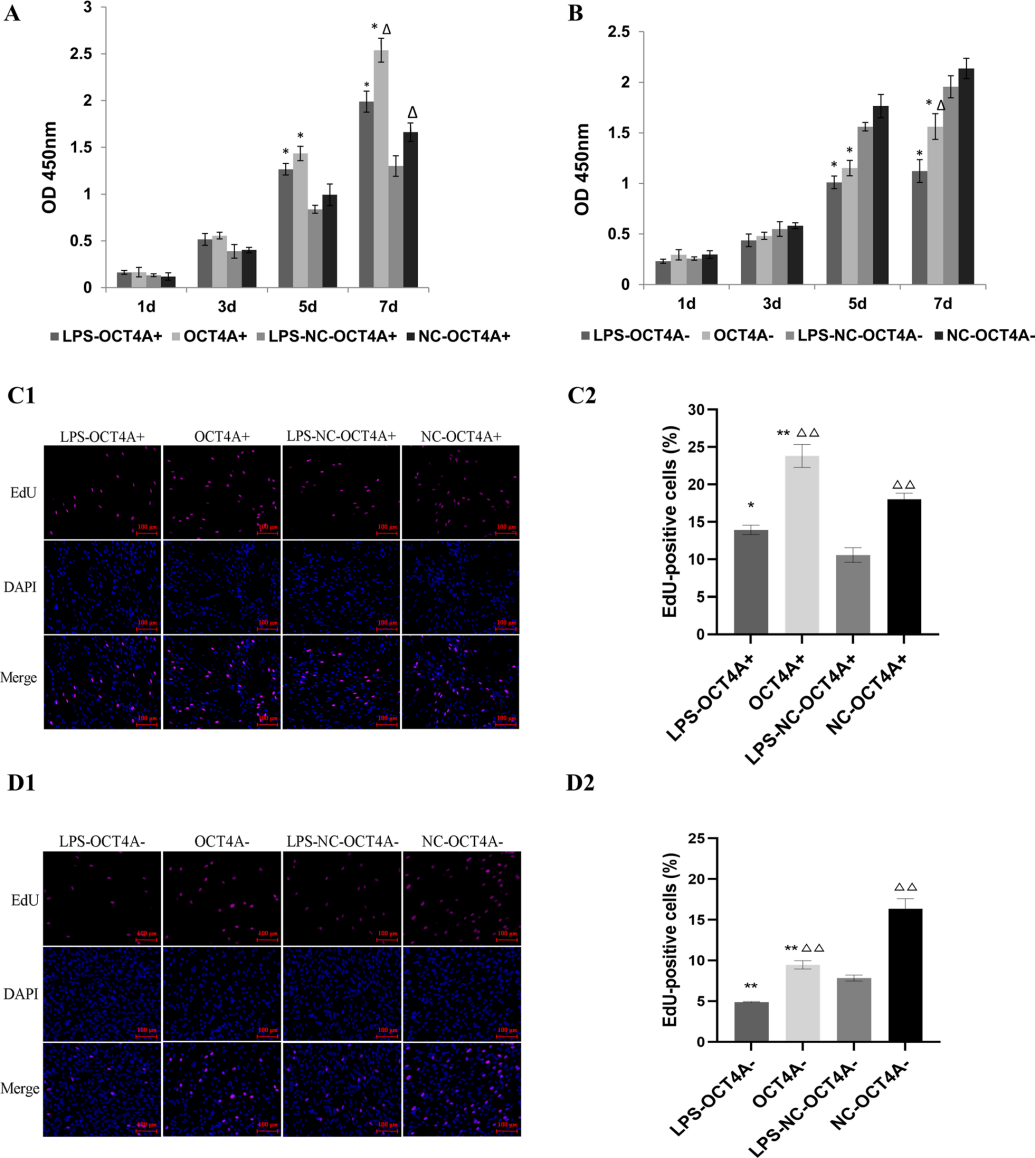
Effect of OCT4A on the prolifeation of hDPSCs. **A**, **B** The CCK-8 assay showed that the inflammatory stimulus with 1 μg/mL LPS significantly inhibited the proliferation of hDPSCs. Overexpression of OCT4A alleviated the changes in cell proliferation caused by LPS stimulation, while knockdown of OCT4A further attenuated the proliferation of hDPSCs. **C**, **D** EdU staining showed that overexpression of OCT4A obviously increased the percentage of EdU-positive cells in LPS-treated hDPSCs, while EdU-positive cells were markedly inhibited by OCT4A interference. *: compared with the vector group, *P* < 0.05, **: compared with the vector group, *P* < 0.01; ∆: compared with the LPS stimulation group, *P* < 0.05; ∆∆: compared with the LPS stimulation group, *P* < 0.01

**Fig. 3:**
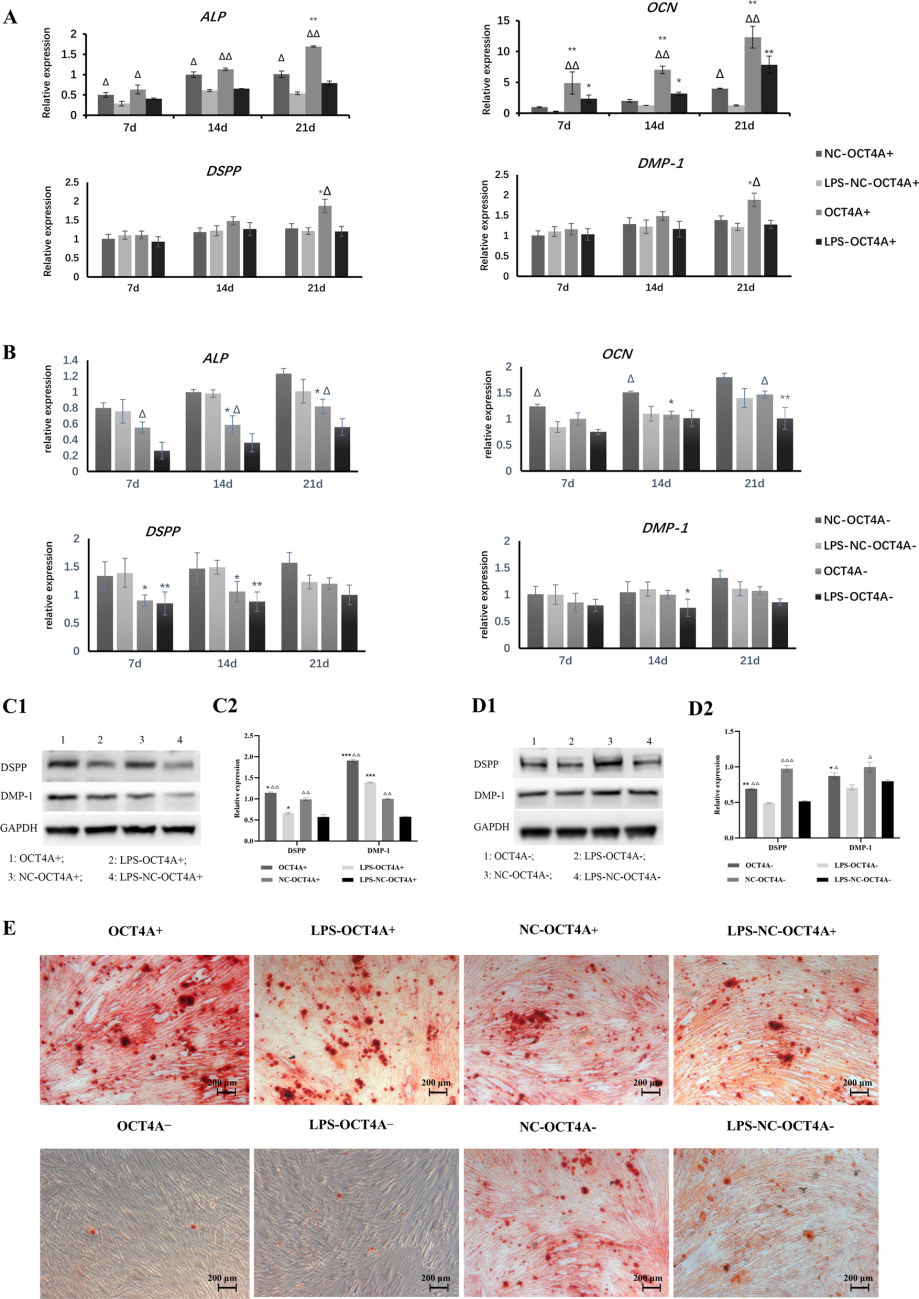
Effect of OCT4A on the odontogenic differentiation of hDPSCs. **A**, **B** After 7, 14, and 21 days of odontogenic induction, LPS reduced the expression of the odontogenic-related genes ALP, OCN, DSPP and DMP-1 compared with the unstimulated cells. Overexpression of OCT4A promoted the expression of these genes in both the LPS-stimulated and nonstimulated groups (A), while knockdown of OCT4A decreased their expression compared with the vector groups (B). **C**, **D** After odontogenic induction for 21 days, the protein expression of DSPP and DMP-1 in each group showed a similar trend to their gene expression (C1 & D1: Representative bands of the protein expression of DSPP and DMP-1; C2 & D2: Quantitative analysis results of western blotting; Full-length blots are presented in Additional file 4). **E** The calcification nodule formation of hDPSCs was shown by Alizarin red staining (× 50). The LPS inflammatory stimulus reduced calcification nodule formation. The number and size of the mineral nodules in DPSCs with OCT4A overexpression were greater than those in the vector groups, while DPSCs with OCT4A knockdown exhibited the opposite result. *: compared with the vector group, *P* < 0.05; **: compared with the vector group, *P* < 0.01; ***: compared with the vector group, *P* < 0.001; ∆: compared with the LPS stimulation group, *P* < 0.05; ∆∆: compared with the LPS stimulation group, *P* < 0.01; ∆∆∆: compared with the LPS stimulation group, *P* < 0.001

**Fig. 4:**
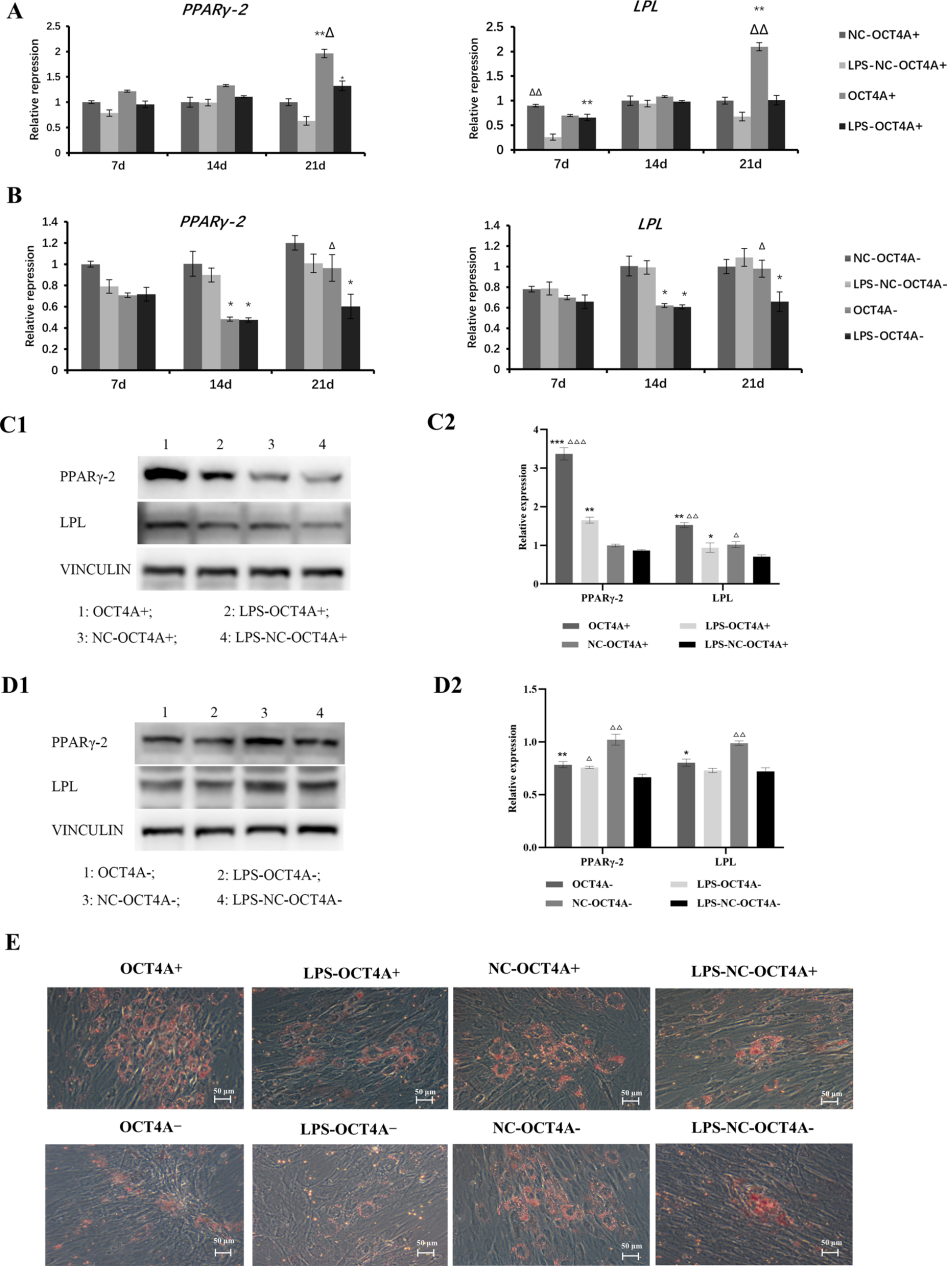
Effect of OCT4A on the adipogenic differentiation of hDPSCs. **A**, **B** After 7, 14, and 21 days of adipogenic induction, LPS reduced the expression of the adipogenic-related genes PPARγ-2 and LPL compared with the unstimulated cells. Overexpression of OCT4A promoted the expression of these genes in both the LPS-stimulated and nonstimulated groups (A), while knockdown of OCT4A decreased their expression compared with the vector groups (B). **C**, **D** After adipogenic induction for 21 days, the protein expression of PPARγ-2 and LPL in each group showed a similar trend to their gene expression (C1 & D1: Representative bands of the protein expression of PPARγ-2 and LPL; C2&D2: Quantitative analysis results of western blotting; Full-length blots are presented in Additional file 4). **E** The lipid droplet formation of hDPSCs was observed by oil red O staining (× 200). The LPS inflammatory stimulus reduced lipid droplet formation. The number and size of the lipid droplets in hDPSCs with OCT4A overexpression were greater than those in the vector groups, while hDPSCs with OCT4A knockdown exhibited the opposite result. *: compared with the vector group, *P* < 0.05; **: compared with the vector group, *P* < 0.01; ***: compared with the vector group, *P* < 0.001; ∆: compared with the LPS stimulation group, *P* < 0.05; ∆∆: compared with the LPS stimulation group, *P* < 0.01; ∆∆∆: compared with the LPS stimulation group, *P* < 0.001

The CCK-8 assay showed that the inflammatory stimulus with 1 μg/mL LPS caused significant downregulation of the proliferation of hDPSCs (Fig. 2A, B). Overexpression of OCT4A partially alleviated the changes in cell proliferation caused by LPS stimulation, while knockdown of OCT4A further inhibited the proliferation of hDPSCs (Fig. 2A, B). Furthermore, overexpression of OCT4A obviously increased the percentage of EdU-positive cells in LPS-treated hDPSCs, while EdU-positive cells were markedly inhibited by OCT4A interference (Fig. 2C, D). After 21 days of odontogenic or adipogenic induction, both the odontogenic and adipogenic differentiation capabilities of hDPSCs in the LPS stimulation groups were attenuated (Figs. 3, 4). Overexpression of OCT4A upregulated the expression of the odontogenic-related markers ALP, OCN, DSPP, and DMP-1 and the adipogenic-related markers PPARγ-2 and LPL at both the gene and protein levels (Figs. 3A, C, Fig. 4A, C), while OCT4A knockdown caused downregulation of these markers compared with expression in the vector groups (Figs. 3B–D and 4B–D). OCT4A overexpressing hDPSCs exhibited increased and larger calcification nodules and lipid droplet formation compared with the normal vector groups, whereas the OCT4A knockdown groups showed the opposite results (Figs. 3E, 4E).

### Microarray expression profile analysis of lncRNAs and mRNAs in OCT4A overexpressing hDPSCs

**Fig. 5:**
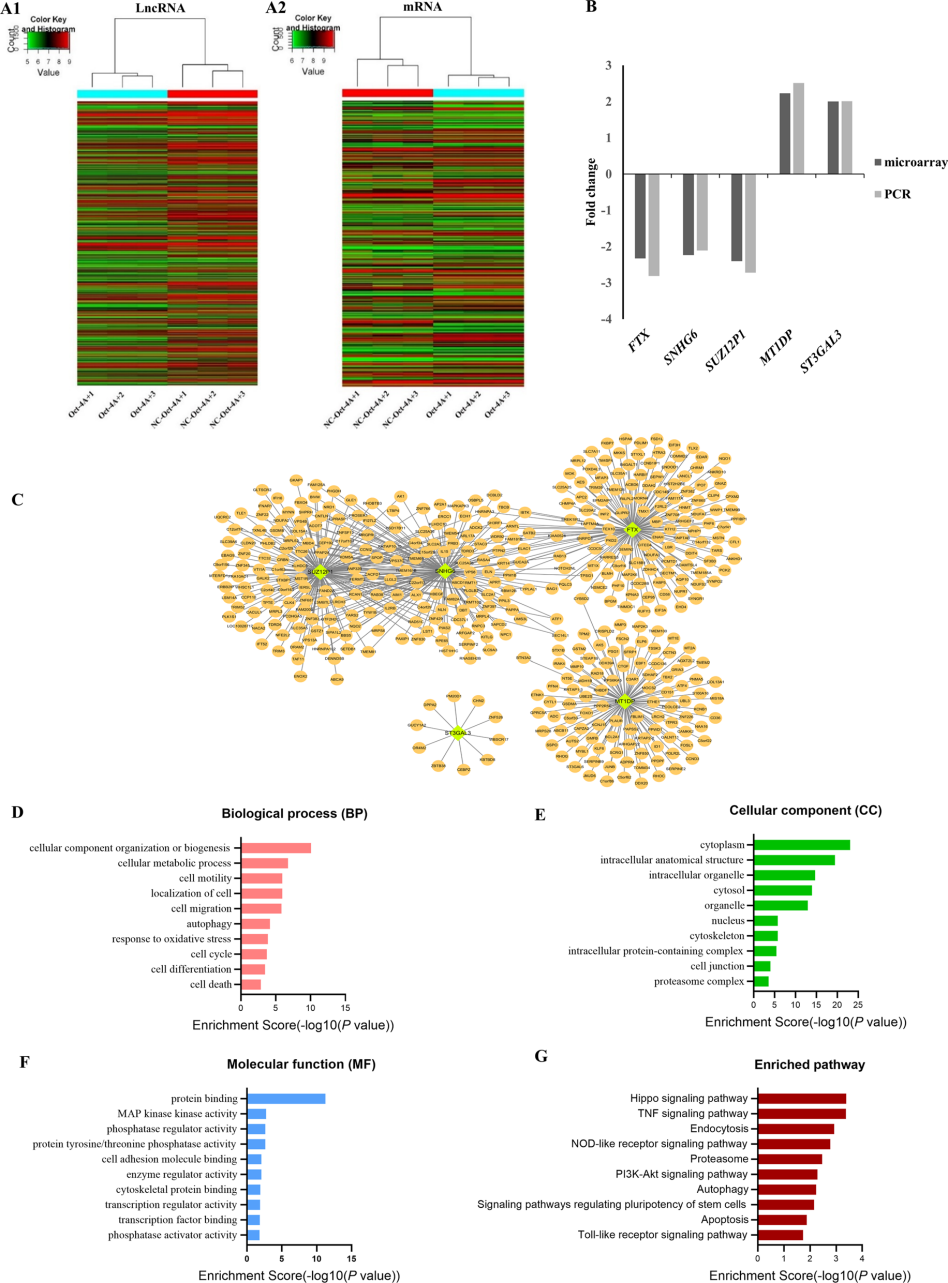
Bioinformatics analysis of differentially expressed lncRNAs between OCT4A-overexpressing and vector hDPSCs. **A** Hierarchical clustering for the expression profiles of differentially expressed lncRNAs and mRNAs between OCT4A-overexpressing and vector hDPSCs. (*red:* high relative expression; *green*: low relative expression). **B** Verification of 5 differentially expressed lncRNAs between OCT4A-overexpressing and vector hDPSCs. **C** Target lncRNAs-differentially expressed mRNAs coexpression network. **D**–**G** GO and KEGG analyses based on CNC analysis results of 5 candidate lncRNAs. **D** GO analysis of coexpressed mRNAs on biological process (BP). **E** GO analysis of coexpressed mRNAs on cellular component (CC). **F** GO analysis of coexpressed mRNAs on molecular function (MF). **G** KEGG analysis of coexpressed mRNAs. GO, Gene Ontology; KEGG, Kyoto Encyclopedia of Genes and Genomes; CNC, coding–noncoding gene coexpression; lncRNA, long non-coding RNA; mRNA, massage RNA

To determine whether lncRNAs were involved in the OCT4A-related network that regulates the self-renewal of hDPSCs, human lncRNA microarrays were used to identify the lncRNA expression profiles in OCT4A-overexpressing and vector hDPSCs. The results (Fig. 5A) showed that 978 lncRNAs (250 of which were upregulated and 728 downregulated) and 404 mRNAs (222 of which were upregulated and 182 downregulated) were potential differentially expressed genes (fold change ≥ 2, *P* < 0.05). The scatter plot in Additional file 2: Fig. S2 shows a strong correlation between samples, and the box plot indicated a similar distribution of the intensities among all samples (Additional file 2: Fig. S2A–D).

GO and KEGG pathways were employed to analyze differentially expressed mRNAs in OCT4A-overexpressing hDPSCs. GO analysis showed that a variety of biological processes, such as extracellular matrix organization and decomposition, system development, protein and macromolecule metabolic process, signal transduction, cellular response to external stimulus, and cell communication (Additional file 3: Fig. S3A, B), were significantly involved*.* KEGG analysis of the differentially expressed mRNAs showed several important pathways such as the Toll-like receptor signaling pathway, TNF signaling pathway and cytokine‒cytokine receptor interaction, were significantly enriched (Additional file 3: Fig. S3C). Functional identification of the differentially expressed mRNAs confirmed that OCT4A may play an important role in the biological processes of hDPSCs through its effects on these signaling pathways under inflammatory conditions.

### Validation of the differentially expressed lncRNAs and CNC network analysis

To verify the microarray results, five of the differentially expressed lncRNAs were selected and analyzed in OCT4A-overexpressing and vector hDPSCs. The qRT‒PCR results were consistent with the microarray data (Fig. 5B). Based on the results of quantitative validation, we performed coexpression analysis and constructed a CNC network (Fig. 5C). Next, we conducted GO and KEGG pathway analyses of differentially expressed mRNAs coexpressed with the above five candidate lncRNAs. The GO analysis showed that differentially expressed mRNAs coexpressed with the candidate lncRNAs were involved in 350 biological processes, 76 cellular components, and 46 molecular functions. The significantly enriched GO terms were cellular component organization/biogenesis, cellular metabolic process, cell motility, cell migration, response to oxidative stress and cell cycle in biological processes and cytoplasm, intracellular anatomical structure, intracellular organelle, cytosol, and cytoskeleton in cellular components (Fig. 5D, E). In addition, the molecular functions of protein binding, MAP kinase kinase activity, cell adhesion molecule binding, transcription regulator activity and transcription factor binding were significantly enriched (Fig. 5F). KEGG pathway analysis showed that differentially expressed mRNAs coexpressed with the candidate lncRNAs were enriched in Hippo signaling pathway, TNF signaling pathway, PI3K-Akt signaling pathway, autophagy and signaling pathways regulating pluripotency of stem cells (Fig. 5G). Functional identification of the coexpressed mRNAs indicated that the candidate lncRNAs may play a potential role in the biological functions of OCT4A-overexpressing hDPSCs through their effects on the coexpressed mRNAs to regulate these biological processes and signaling pathways.

### OCT4A repressed lncRNA FTX expression in hDPSCs

**Fig. 6:**
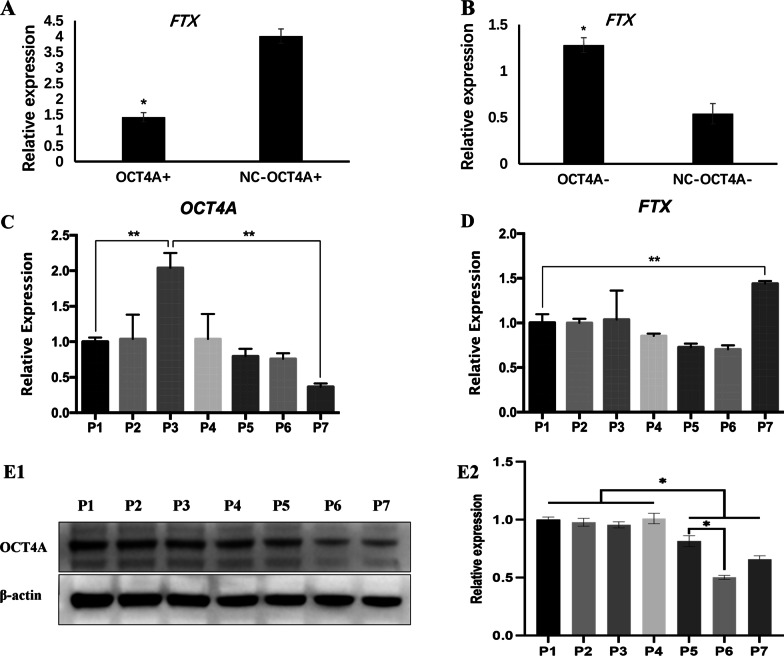
Verification of the identity of differentially expressed lncRNAs.** A**, **B** Expression of FTX in OCT4A-overexpressing or OCT4A-knockdown hDPSCs (*: compared with the vector group, *P* < 0.05). **C**, **D** Gene expression profiles of OCT4A and FTX in different cell passages of hDPSCs (*: *P* < 0.05, **: *P* < 0.01). **(E)** Protein expression of OCT4A in different cell passages of hDPSCs (E1: Representative bands of the protein expression of OCT4A; E2: Quantitative analysis results of western blotting; Full-length blots are presented in Additional file 4, *: *P* < 0.05)

Among the five lncRNAs, FTX (fold change = 2.81, *P* < 0.05) was the most differentiated lncRNA from the verification results (Fig. 5B), and was taken as the candidate lncRNA. The real-time PCR results confirmed that FTX was downregulated in OCT4A-overexpressing hDPSCs and upregulated in OCT4A-knockdown hDPSCs, which was consistent with the microarray analyses (Fig. 6A, B). In addition, FTX and OCT4A exhibited an opposing expression trend in subsequent passages of hDPSCs. The PCR and western blotting results showed that OCT4A was activated at early passages (P1–P3) and then subsequently decreased with extended passages of hDPSCs (Fig. 6C, E), while FTX showed an expression profile that was downregulated from P1 to P6 and upregulated at P7 (Fig. 6D), suggesting their different roles in regulating cell fate.

### Overexpression of FTX inhibited proliferation and multidifferentiation of hDPSCs under LPS stimulation

**Fig. 7:**
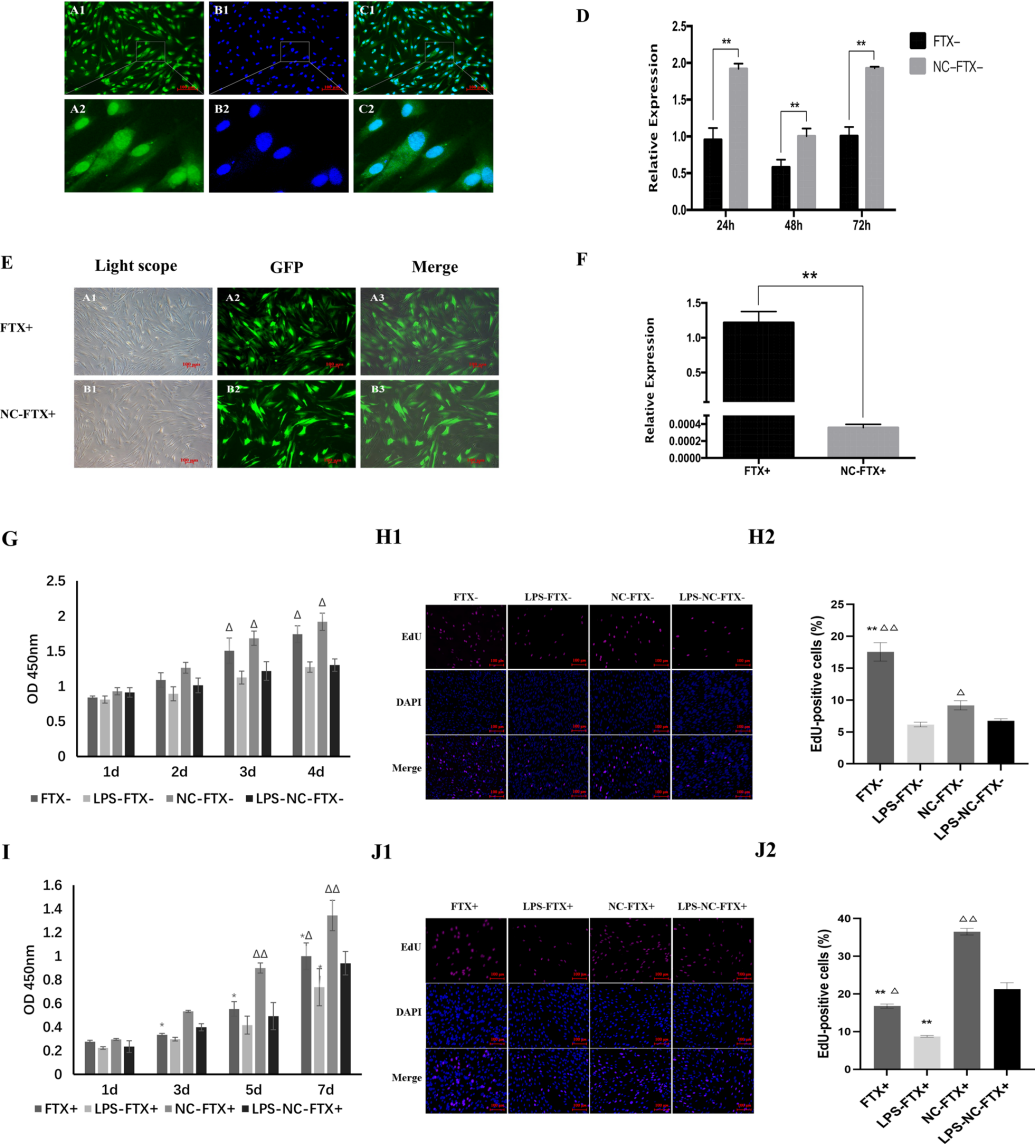
Establishment of FTX+ /hDPSCs or FTX-/hDPSCs and the effect of FTX on cell proliferation. **A**–**C** The subcellular localization of FTX in hDPSCs was detected by fluorescence in situ hybridization (× 200). (A1, A2: The green fluorescence of the FTX probes; B1, B2: Nucleus stained with DAPI; C1, C2: Merged pictures). **D** FTX expression at different time points after FTX interference in hDPSCs. **E** Green fluorescence of FTX overexpression lentivirus in hDPSCs (× 50). **F** FTX expression after overexpression in hDPSCs. **G**, **I** The CCK-8 assay showed that the LPS stimulus significantly inhibited the proliferation of hDPSCs. Knockdown of FTX promoted cell proliferation, while overexpression of FTX further reduced the proliferation of hDPSCs in the LPS groups. **H**, **J** The EdU assay showed that FTX silencing conspicuously promoted the incorporation of EdU in the genome of LPS-induced hDPSCs, while upregulation of FTX notably decreased the EdU-positive cells. *: compared with the vector group, *P* < 0.05; **: compared with the vector group, *P* < 0.01; ∆: compared with the LPS stimulation group, *P* < 0.05; ∆∆: compared with the LPS stimulation group, *P* < 0.01

We next treated hDPSCs with FTX overexpression lentivirus for stable transfection or human-FTX Smart Silencer for transient transfection to clarify the function of FTX. The FISH results revealed that FTX was distributed both in the nucleus and cytoplasm of hDPSCs (Fig. 7A–C). Figure 7D&F showed that the FTX expression was effectively reduced in hDPSCs transfected with human-FTX Smart Silencer, but significantly enhanced in FTX-overexpressing hDPSCs. As shown in Fig. 7G, I, 1 μg/mL LPS suppressed the proliferation of hDPSCs. Overexpression of FTX further reduced the proliferation activity of hDPSCs (*P* < 0.05). In addition, the EdU assay results showed that FTX silencing conspicuously promoted the incorporation of EdU in the genome of LPS-induced hDPSCs, while upregulation of FTX notably decreased the EdU-positive cells (Fig. 7H, J).

**Fig. 8:**
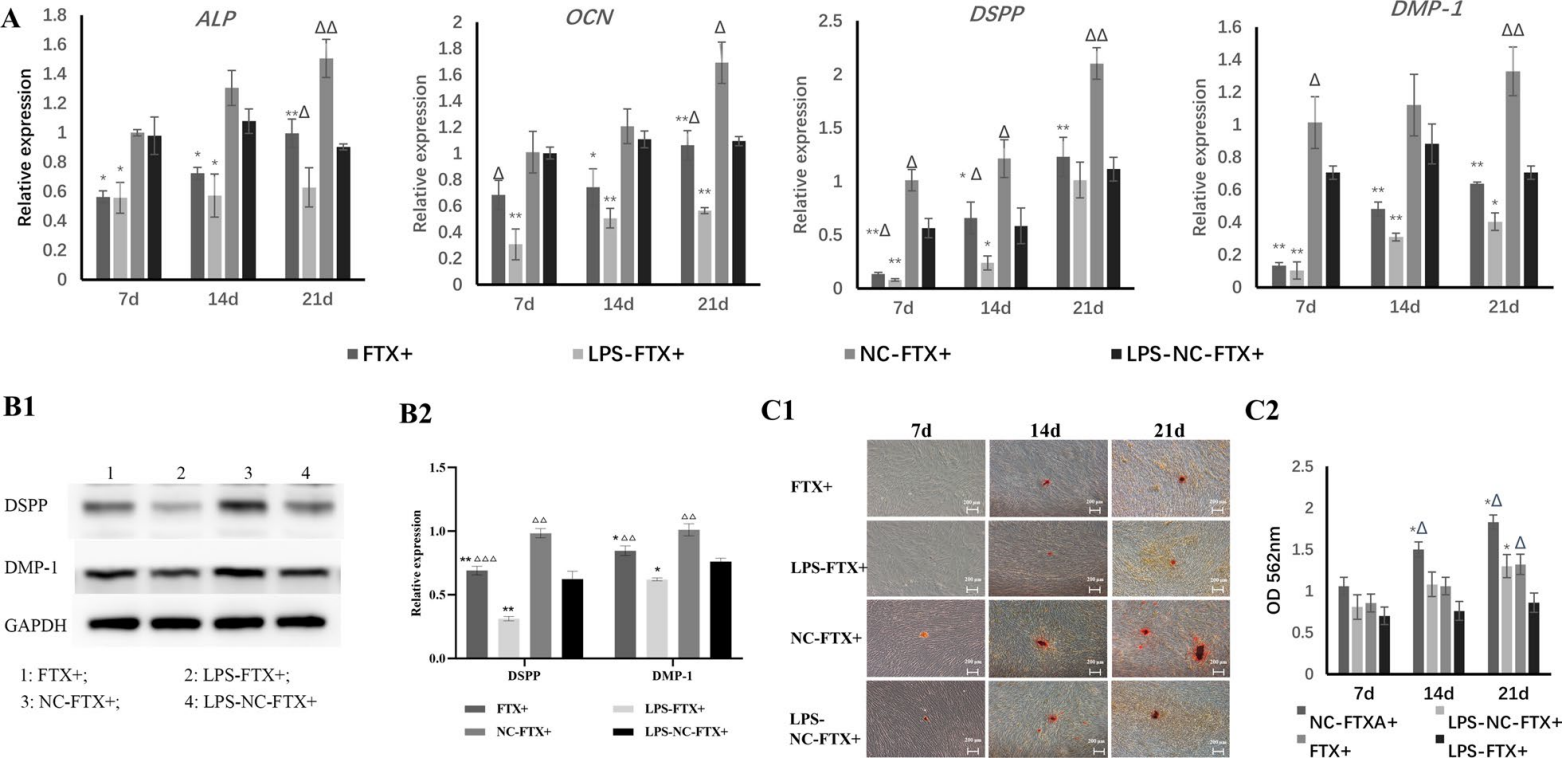
Effect of FTX on the odontogenic differentiation of hDPSCs. **A** After 21 days of odontogenic induction, the expression of the odontogenic-related genes ALP, OCN, DSPP and DMP-1 was downregulated in the LPS groups compared with the unstimulated groups, as well as in the FTX-overexpressing groups compared with the vector groups. Overexpression of FTX further decreased their expression in the LPS groups. **B** After odontogenic induction for 21 days, the protein expression of DSPP and DMP-1 in each group showed a similar trend to their gene expression (B1: Representative bands of the protein expression of DSPP and DMP-1; B2: Quantitative analysis results of western blotting; Full-length blots are presented in Additional file 4). **C** The calcification nodule formation of hDPSCs overexpressing FTX is shown by Alizarin red staining. (C1) Inflammatory stimulation with LPS inhibited calcification nodule formation. The number and size of the mineral nodules in DPSCs with FTX overexpression were lower than those in the vector groups (× 50). (C2) The OD value of the mineralization nodules in hDPSCs overexpressing FTX was significantly downregulated compared with that in the vector groups at each time point. *: compared with the vector group, *P* < 0.05; **: compared with the vector group, *P* < 0.01; ∆: compared with the LPS stimulation group, *P* < 0.05; ∆∆: compared with the LPS stimulation group, *P* < 0.01; ∆∆∆: compared with the LPS stimulation group, *P* < 0.001

**Fig. 9:**
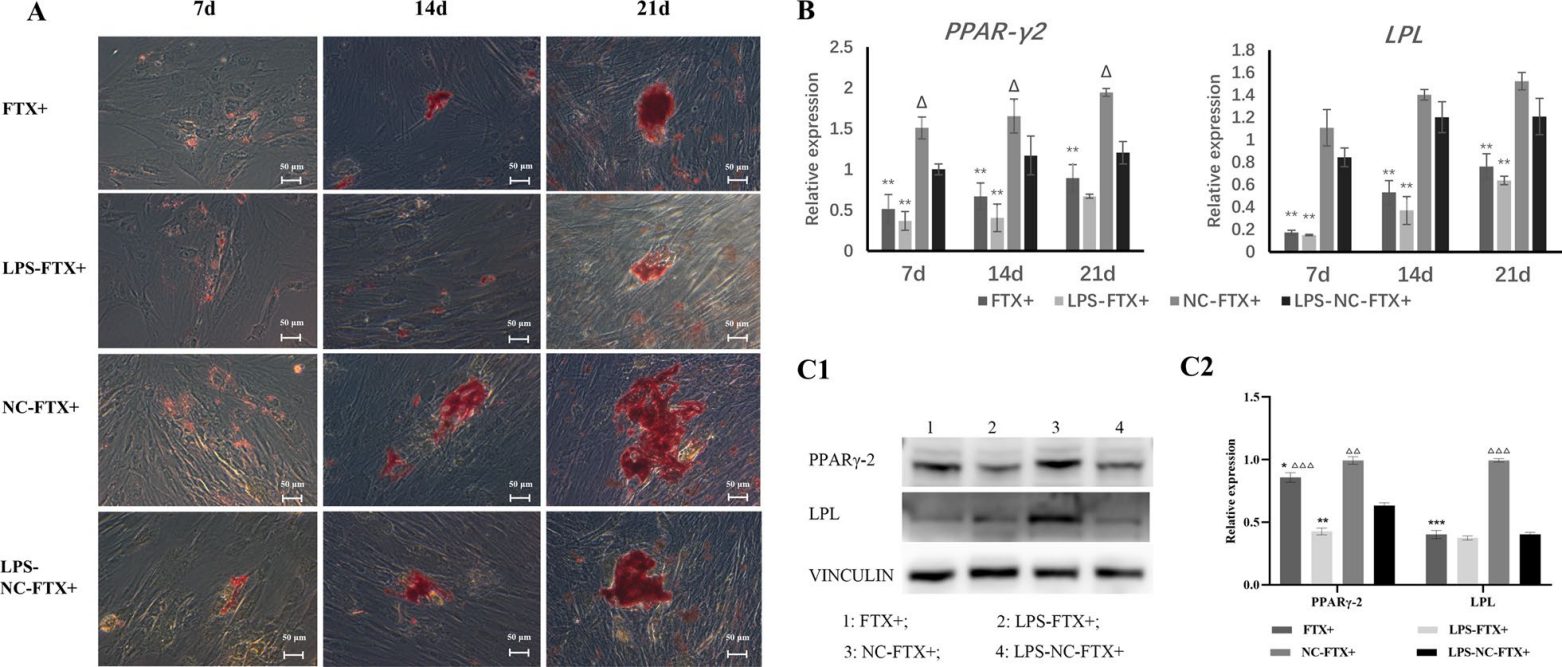
Effect of FTX on the adipogenic differentiation of hDPSCs. **A** After 21 days of adipogenic induction, lipid droplet formation in hDPSCs was observed by oil red O staining (× 200). The LPS inflammatory stimulus reduced lipid droplet formation. DPSCs overexpressing FTX exhibited fewer and smaller lipid droplets than the vector groups. **B** The real-time PCR results showed that LPS reduced the expression of the adipogenic-related genes PPARγ-2 and LPL, and overexpression of FTX further decreased their expression in both the LPS-stimulated and nonstimulated groups. **C** The protein expression of PPARγ-2 and LPL in each group showed a similar trend to their gene expression. (C1: Representative bands of the protein expression; C2: Quantitative analysis of western blotting; Full-length blots are presented in Additional file 4). *: compared with the vector group, *P* < 0.05; **: compared with the vector group, *P* < 0.01; ***: compared with the vector group, *P* < 0.001; ∆: compared with the LPS stimulation group, *P* < 0.05; ∆∆: compared with the LPS stimulation group, *P* < 0.01; ∆∆∆: compared with the LPS stimulation group, *P* < 0.001

After 21 days of induction, the 1 μg/mL LPS inflammatory stimulus significantly reduced the odontogenic and adipogenic differentiation capabilities of hDPSCs (Figs. 8, 9). Overexpression of FTX further suppressed the expression of the odontogenic-related markers ALP, OCN, DSPP, and DMP-1 as well as the adipogenic-related markers PPARγ-2 and LPL at both the gene and protein levels at each time point (Figs. 8, 9). The size and number of the mineral nodules or lipid droplets in DPSCs with FTX overexpression were smaller and less than those in the vector groups (Figs. 8C, 9A).

### OCT4A inhibits lncRNA FTX transcriptional levels by binding to its promoter region

**Fig. 10:**
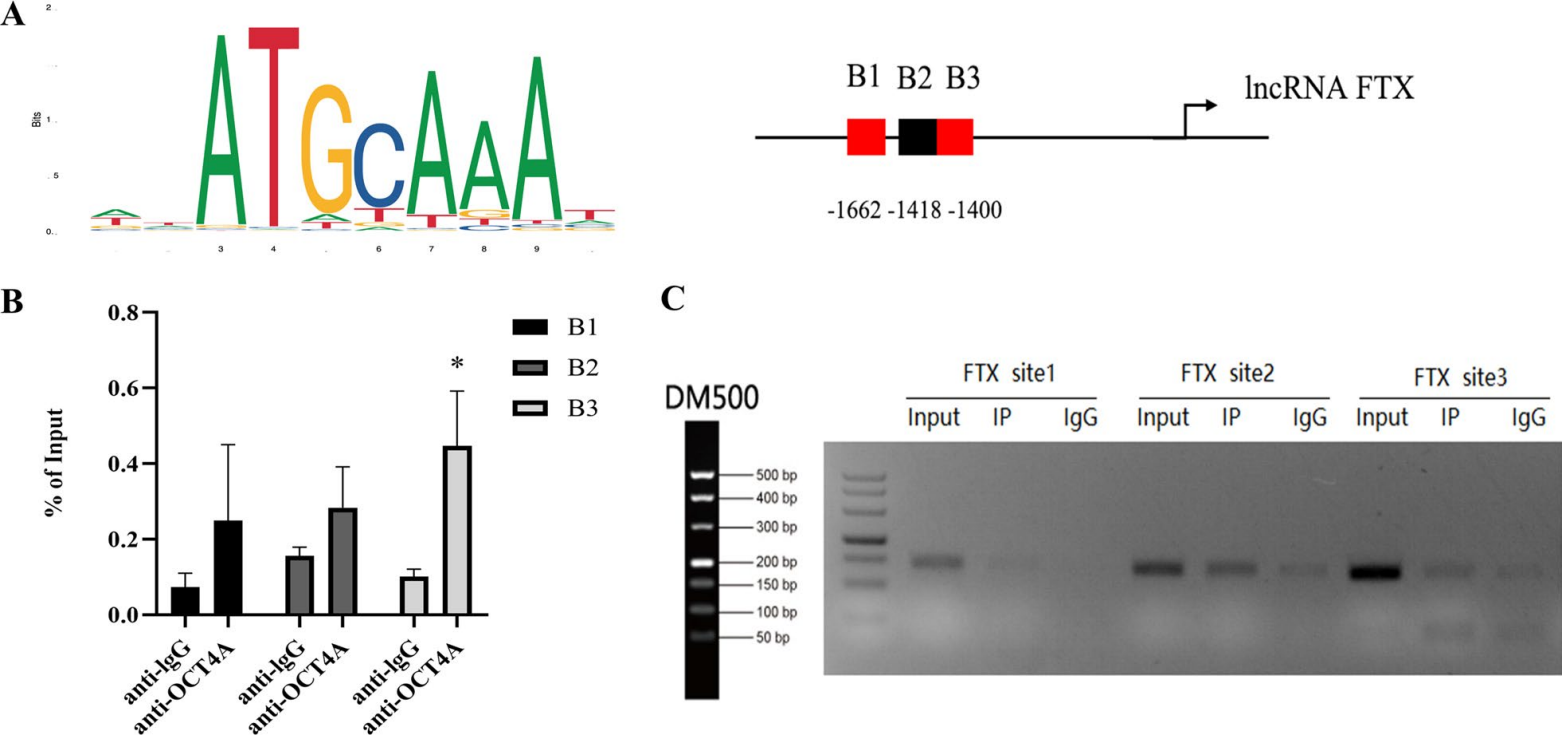
OCT4A binds to the promoter region of lncRNA FTX to inhibit its transcription in hDPSCs. **A** The Jaspar and PROMO algorithms were used to analyze 3 potential binding sites of OCT4A on the lncRNA FTX promoter. **B** ChIP‒qPCR analysis of OCT4A occupancy in the lncRNA FTX promoter in hDPSCs. **C** PCR products were analyzed by agarose gel electrophoresis (Full-length gels are presented in Additional file 4. *: compared with the lgG group,* P* < 0.05)

We further explored the potential mechanism by which OCT4A downregulated the expression of lncRNA FTX. The online TF prediction tools JASPAR (http://jaspar.genereg.net) and PROMO (http://alggen.lsi.upc.es/cgibin/promo_v3/promo/promoinit.cgi?dirDB=TF_8.3) were used to predicate that several TFs, including OCT4A, may bind to the conserved octamer motif (ATGCAAAT) in the promoter region of lncRNA FTX, and 3 potential binding sites of OCT4A were analyzed (Fig. 10A). To determine which OCT4A binding site was responsive to OCT4A-mediated transcriptional repression of FTX, a ChIP assay was performed. Our results confirmed that the predicted binding site 3 (B3) of lncRNA FTX promoter DNA fragments presented notably higher binding affinity with anti-OCT4A antibody, compared with the isotype control antibody (Fig. 10B, C). Overall, these data demonstrated that OCT4A inhibited the transcription of lncRNA FTX by binding to its promoter.

### FTX suppressed the expression of OCT4A and its downstream pluripotent transcription factors in hDPSCs

**Fig. 11:**
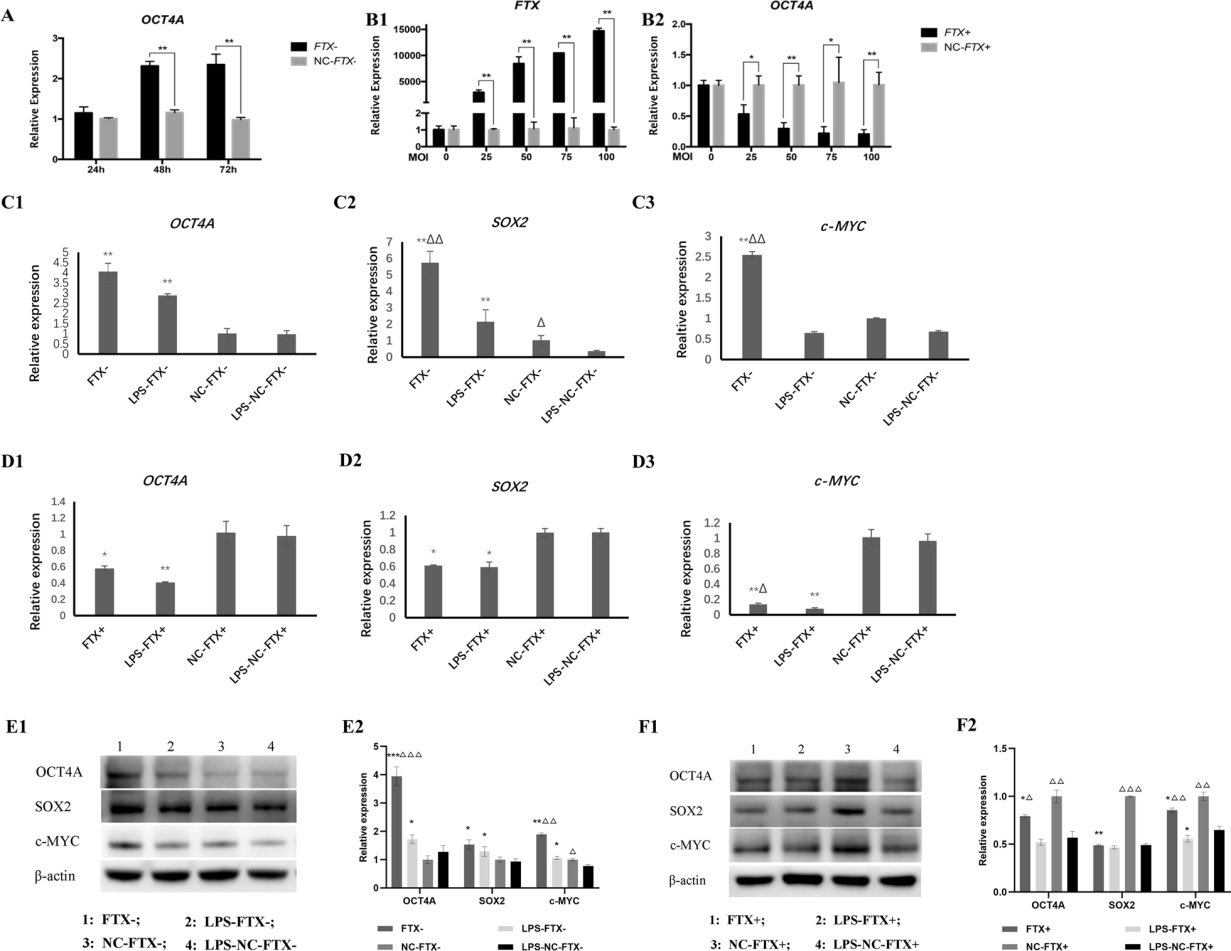
Effect of FTX on the expression of OCT4A and other pluripotent transcription factors in hDPSCs. **A** Expression of the OCT4A gene was upregulated after the interference of FTX expression in hDPSCs for 48 and 72 h. **B** Expression of the OCT4A gene showed a gradually declining trend in hDPSCs treated with an increasing MOI of FTX overexpression lentivirus. **C**–**F** Expression of OCT4A, SOX2 and c-MYC in FTX-knockdown or FTX-overexpressing hDPSCs. (C&D: mRNA expression; E1&F1: Representative bands of the protein expression; E2&F2: Quantitative analysis results of western blotting; Full-length blots are presented in Additional file 4; *: compared with the vector group, *P* < 0.05; **: compared with the vector group, *P* < 0.01; ***: compared with the vector group, *P* < 0.001; ∆: compared with the LPS stimulation group, *P* < 0.05; ∆∆: compared with the LPS stimulation group, *P* < 0.01; ∆∆∆: compared with the LPS stimulation group, *P* < 0.001

To explore how FTX might integrate into the core transcriptional regulatory networks of the self-renewal ability of hDPSCs, the effect of FTX on the expression of the pluripotent transcription factor OCT4A and its downstream pluripotent transcription factors SOX2 and c-MYC was investigated. After interfering with FTX expression for 48 and 72 h, the expression of OCT4A was significantly upregulated (Fig. 11A). Conversely, in the FTX-overexpressing groups, with increasing virus concentration, the expression of OCT4A was downregulated and showed a gradually declining trend (Fig. 11B). The mRNA expression of the pluripotent transcription factors OCT4A, SOX2 and c-MYC was suppressed under LPS inflammatory stimulation compared to that in the untreated groups. FTX knockdown improved the expression of OCT4A, SOX2 and c-MYC in hDPSCs treated with LPS or not. In contrast, overexpression of FTX reduced the expression of OCT4A, SOX2 and c-MYC (Fig. 11C, D). Western blot analysis showed similar trends (Fig. 11E, F).

## Discussion

DPSCs, a kind of adult stem cell in dental pulp tissues, are promising candidates for pulp regeneration or other regenerative therapies. When irritated by bacteria or their toxic components, pulp immune cells will initiate an early inflammatory response to eliminate invading microbes. DPSCs will consequently migrate to the damaged site and differentiate into odontoblasts to synthesize reparative dentin, initiating pulp repair and regeneration [2, 3, 17]. However, due to the specific structure of dental pulp which is surrounded by rigid tissues, the antibacterial activities can easily induce an inflammatory cascade to propagate sustained inflammation throughout the pulp [18–22], which tremendously damages vital pulp tissue and irreversibly impedes the repair responses of DPSCs, eventually leading to pulp necrosis. Hence, facilitating the self-renewal capacity of DPSCs while ameliorating the inflammatory microenvironment will significantly contribute to the self-repair of dental pulp.

OCT4A, the most important transcription factor located at the centre of the reprogramming network, serves as the key regulator for the self-renewal and pluripotency maintenance of ESCs [23]. We previously reported that OCT4A improved the proliferation and odontogenic differentiation of hDPCs [8], indicating the potential for OCT4A to regulate pulp repair. In the present study, we first detected the effects of OCT4A on the pluripotency property of hDPSCs in an inflammatory microenvironment. Our results showed that the proliferation and odontogenic and adipogenic differentiation capacities of hDPSCs were significantly attenuated by the LPS inflammatory stimulus. Overexpression of OCT4A alleviated the changes in cell proliferation and multidifferentiation capacities caused by LPS stimulation, while knockdown of OCT4A further inhibited these properties, suggesting the crucial role of OCT4A in maintaining the pluripotency of hDPSCs in inflammatory conditions.

The circuitry controlling self-renewal in pluripotent cells has a complex interplay between pluripotent transcription factors, lncRNAs and chromatin modifying complexes [12]. A search for OCT4 binding sites in lncRNAs that are expressed in mouse ESCs, revealed the recognition of 10% of the OCT4 binding sites in the vicinity of lncRNA genes [24]. Further studies demonstrated that diverse lncRNAs, such as Gomafu [24], Panct-1 [25], and VLDLR [26], acted in concert with OCT4A to regulate the pluripotency of ESCs. To verify our hypothesis that OCT4A may promote the repair of injured pulp by interacting with lncRNAs, microarray expression profiles were utilized to identify the differentially expressed lncRNAs and mRNAs in OCT4A overexpressing and vector hDPSCs. A total of 978 differentially expressed lncRNAs and 404 differentially expressed mRNAs were identified. Five lncRNAs were selected for validation and the PCR results supported the microarray data. In terms of the expression level, the most intriguing lncRNA appeared to be FTX. Further verification results showed that FTX was downregulated in OCT4A overexpressing hDPSCs, but upregulated in OCT4A-knockdown hDPSCs, demonstrating that OCT4A suppressed the expression of FTX.

Human lncRNA FTX, a transcript localized at chromosome X q13.2, has been well conserved during evolution, indicating its vital roles in human biogenic activities [27–30]. Current studies on FTX have mainly focused on its regulation of X chromosome inactivation [31] and the progression of various malignant tumors [32], while its role in pluripotency regulation of stem cells has not yet been reported. FTX was proven to promote the malignant progression of colorectal cancer by targeting the miR-214-5p-JAG1 axis [33] or directly binding to miR-192-5p [34]. Another studies discovered that FTX could inhibit the proliferation and metastasis of lung cancer cells by activating FOXA2 [35] or could suppress the proliferation of hepatocellular carcinoma cells by impeding DNA replication [29]. The different roles of FTX indicate its unique regulation in various tumor cells. Recent studies have also shown that FTX participates in the apoptosis of neurons or cardiomyocytes [36, 37] and regulates the angiogenesis in stroke [38] or hypoxia/reoxygenation-induced cardiomyocyte injury [39], suggesting that FTX may be involved in the apoptosis of adult cells and the repair of tissue injury.

To study the correlation of FTX with the alteration of pluripotency of hDPSCs and whether FTX might participate in the OCT4A-related regulatory network, we preliminarily observed the expression patterns of FTX and OCT4A in cell passages. OCT4A was upregulated at P3 and decreased afterward, while FTX was downregulated from P1 to P6 and upregulated at P7. In our previous study, OCT4A displayed decreased expression during cell passages, which was strongly related to the loss of stemness of hDPCs [8]. The reverse expression profile of FTX and OCT4A in the present study indicated the different roles of OCT4A and FTX in pluripotency maintenance during cell passages. We next explored the effects of FTX on cell function by establishing FTX-overexpressing or FTX-knockdown hDPSCs. In contrast to OCT4A, the proliferation capacity of hDPSCs was further reduced in the FTX-overexpressing group compared with the vector group in the inflammatory microenvironment. Overexpression of FTX also inhibited the expression of odontogenic- and adipogenic-related markers and reduced calcification nodules and lipid droplet formation. These results demonstrated for the first time that FTX suppressed both the proliferation and multidifferentiation capacities of hDPSCs, suggesting its opposite effects on hDPSCs compared with OCT4A. Combined with the microarray verification results, these functional data of FTX clarified that OCT4A contributed to the pluripotency maintenance of hDPSCs by inhibiting FTX expression.

It would be interesting to further investigate the mechanism by which OCT4A regulates FTX. Evidence indicates that lncRNAs can be transcriptionally activated or suppressed by pluripotency transcription factors and can act as molecular mediators of gene expression that determine the pluripotent state of stem cells [24, 40]. In the present study, bioinformatics analysis with online TF prediction databases provided a promising probability that OCT4A may bind to the conserved octamer motif in the promoter region of FTX. ChIP assays further confirmed the enrichment of OCT4A on the predicted binding site 3 (B3) of the lncRNA FTX promoter compared with the isotype control antibody, demonstrating that OCT4A negatively regulated FTX expression by binding to the promoter of FTX and inhibiting its transcription. Hence, our results provide the first evidence that OCT4A promotes the pluripotency of hDPSCs by transcriptionally repressing FTX.

**Fig. 12:**
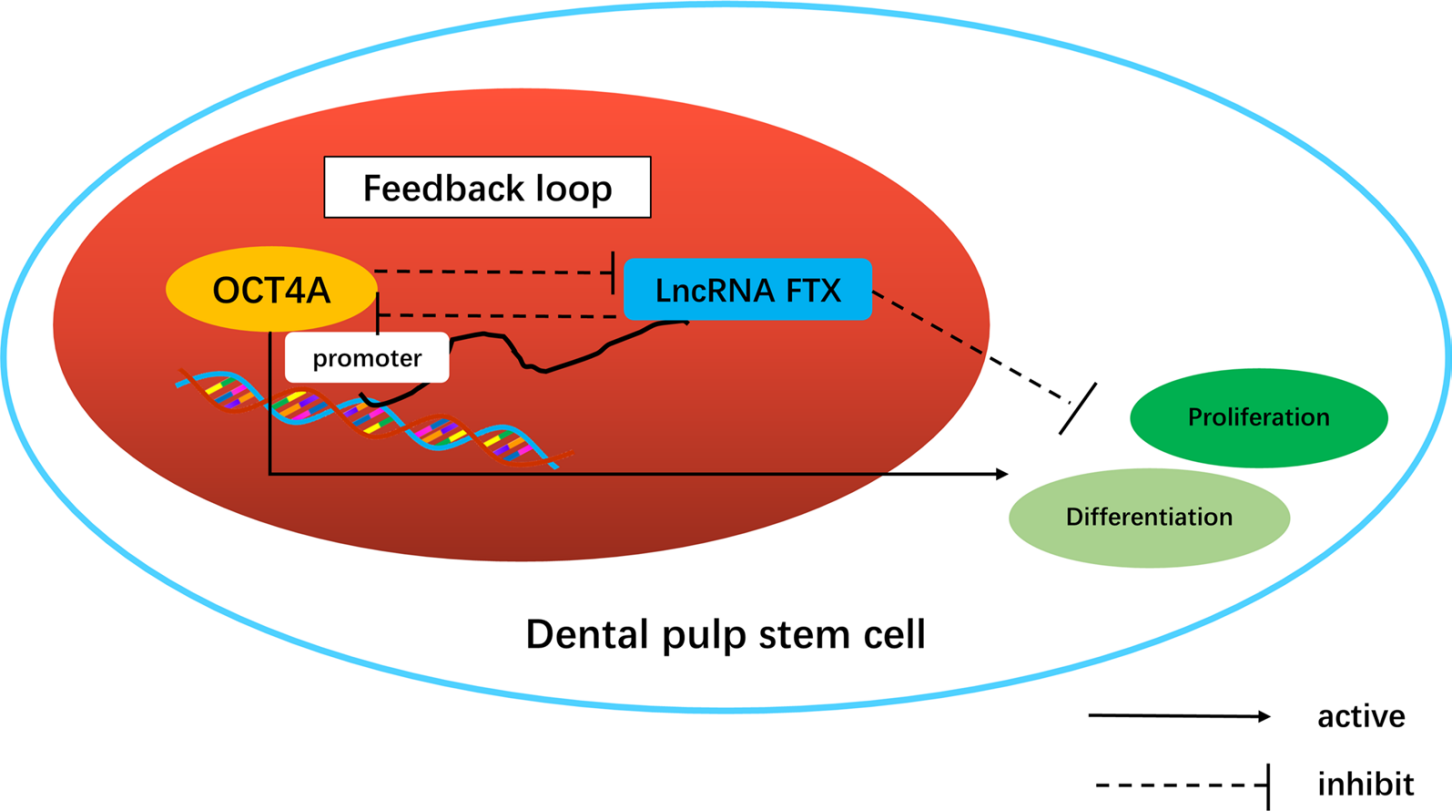
Schematic diagram of the research hypothesis

There is a complex, coordinated orchestra of transcription factors, lncRNAs, chromatin regulators and signal transducer factors in pluripotent cells that regulates the balance between self-renewal and differentiation [41]. Several lncRNAs, such as Linc-RoR [26, 42], GAS5 [43], and Snhg3 [44] are preferentially expressed in ESCs and interact with the core transcription factor network to safeguard pluripotency. They can be activated or repressed by their upstream regulators OCT4, NANOG, SOX2, etc*.*, and in turn regulate the expression of these transcription factors through feedback loops, thus maintaining a pluripotent state or promoting differentiation under certain conditions [12, 41]. Accordingly, we attempted to detect whether FTX altered the expression of OCT4A to implicate it in the core pluripotency regulation of hDPSCs. Interestingly, the results showed that OCT4A was significantly upregulated in FTX-knockdown hDPSCs, whereas it exhibited an obviously and gradually declining trend with increasing concentrations of lentivirus for FTX overexpression, indicating the downregulating effect of FTX on OCT4A expression. Studies have revealed that OCT4A could initiate cell reprogramming through interaction with other pluripotent transcription factors such as SOX2, c-MYC and KLF4, which are critical for preserving stem cell phenotype and responsible for the stemness properties and pluripotency commitment of DPSCs [1, 45, 46]. Our previous research also proved that OCT4A promoted the expression of the downstream markers SOX2 and c-MYC to improve the pluripotency of hDPCs [8]. Therefore, we further investigated the effect of FTX on pluripotent transcription factor expression. The mRNA and protein levels of OCT4A, SOX2 and c-MYC were suppressed in FTX-overexpressing hDPSCs treated with LPS or not, while FTX knockdown showed contrary results, supporting that FTX may inhibit the pluripotency of hDPSCs by decreasing the expression of OCT4A and its downstream pluripotent transcription factors. Taken together, the results of the present study demonstrated a reciprocal inhibition between the expression of FTX and OCT4A in hDPSCs, suggesting that FTX may also be involved in a negative feedback loop with its regulator OCT4A to maintain its level in hDPSCs and eventually regulate the pluripotency homeostasis of hDPSCs (Fig. 12), the specific mechanism of which needs to be further investigated.

## Conclusions

In conclusion, the present study provides a novel evidence that OCT4A plays a vital role in enhancing the self-renewal of hDPSCs by negatively targeting the transcription of lncRNA FTX in an inflammatory microenvironment. Moreover, this study for the first time proposed the negative regulation of FTX in the pluripotency and multilineage differentiation capacity of hDPSCs, expanding our understanding of this particular lncRNA by implicating it in regulatory networks of adult stem cells. The hierarchical organization and possible feedback regulation between key pluripotent transcription factors and lncRNAs might be crucial to uncovering the self-renewal mechanism of inflammatory dental pulp, suggesting novel prospective targets for optimizing dental-derived stem cell sources for regenerative endodontics.

## Supplementary Information

**Additional file 1: Fig. S1**. The morphology and characterization of hDPSCs.Morphology of hDPSCs form primary culturesand the fifth passagein vitro.The expression of surface antigensin hDPSCs were detected by flow cytometry.The osteogenic, adipogenic and chondrogenic differentiation capacity of hDPSCs were verified by Alizarin red, oil red O and Alcian blue staining.

**Additional file 2: Fig. S2**. The changes in expression profiling of lncRNAsand mRNAsin OCT4A-overexpressing and vector hDPSCs.Box plots, which permit the visualization of the dataset distributions.Scatter plots, which are convenient to visualize the variation in gene expression in OCT4A-overexpressing and vector hDPSCs.

**Additional file 3: Fig. S3**. GO and KEGG analyses of differentially expressed mRNAs in OCT4A-overexpressing and vector hDPSCs.GO analysis of differentially expressed mRNAs on biological process.Pathway analysis of differently expressed genes. GO, Gene Ontology; KEGG, Kyoto Encyclopedia of Genes and Genomes.

**Additional file 4**: Uncropped gel/blot images. Uncropped gel/blot images are attached. Images used in the main figure are marked in red squares.

## Data Availability

The microarray data reported in this study has been deposited in NCBI’s Gene Expression Omnibus (GEO) and are accessible through GEO Series accession number GSE225345 (web links: https://www.ncbi.nlm.nih.gov/geo/query/acc.cgi?acc=GSE225345). All other data needed to evaluate the conclusions are present in the paper and/or the Additional files.

## Abbreviations

hDPSCs: Human dental pulp stem cells
hDPCs: Human dental pulp cells
BMSC: Bone marrow mesenchymal stem cells
ESCs: Embryonic stem cells
OCT4A: Octamer-binding transcription factor 4A
lncRNA: Long non-coding RNA
ChIP: Chromatin immunoprecipitation
CCK-8: Cell counting kit-8
ALP: Alkaline phosphatase
OCN: Osteocalcin
DSPP: Dentin sialophosphoprotein
DMP-1: Dentin matrix protein-1
PPARγ-2: Peroxisome proliferator-activated receptor-gamma 2
LPL: Lipoprotein lipase
GO: Gene ontology
KEGG: Kyoto encyclopedia of genes and genomes
CNC: Coding-noncoding gene coexpression
LPS: Lipopolysaccharide
GFP: Green fluorescent protein
DAPI: 4′,6-Diamidino-2-phenylindole
EdU: 5-Ethynyl-2ʹ-deoxyuridine
