# Supplementary figures and images for: The pluripotent factor OCT4A enhances the self-renewal of human dental pulp stem cells by targeting lncRNA FTX in an LPS-induced inflammatory microenvironment
Source: Stem Cell Res Ther. 2023 Apr 27;14:109. doi: 10.1186/s13287-023-03313-8 (PMC10142416; doi:10.1186/s13287-023-03313-8)

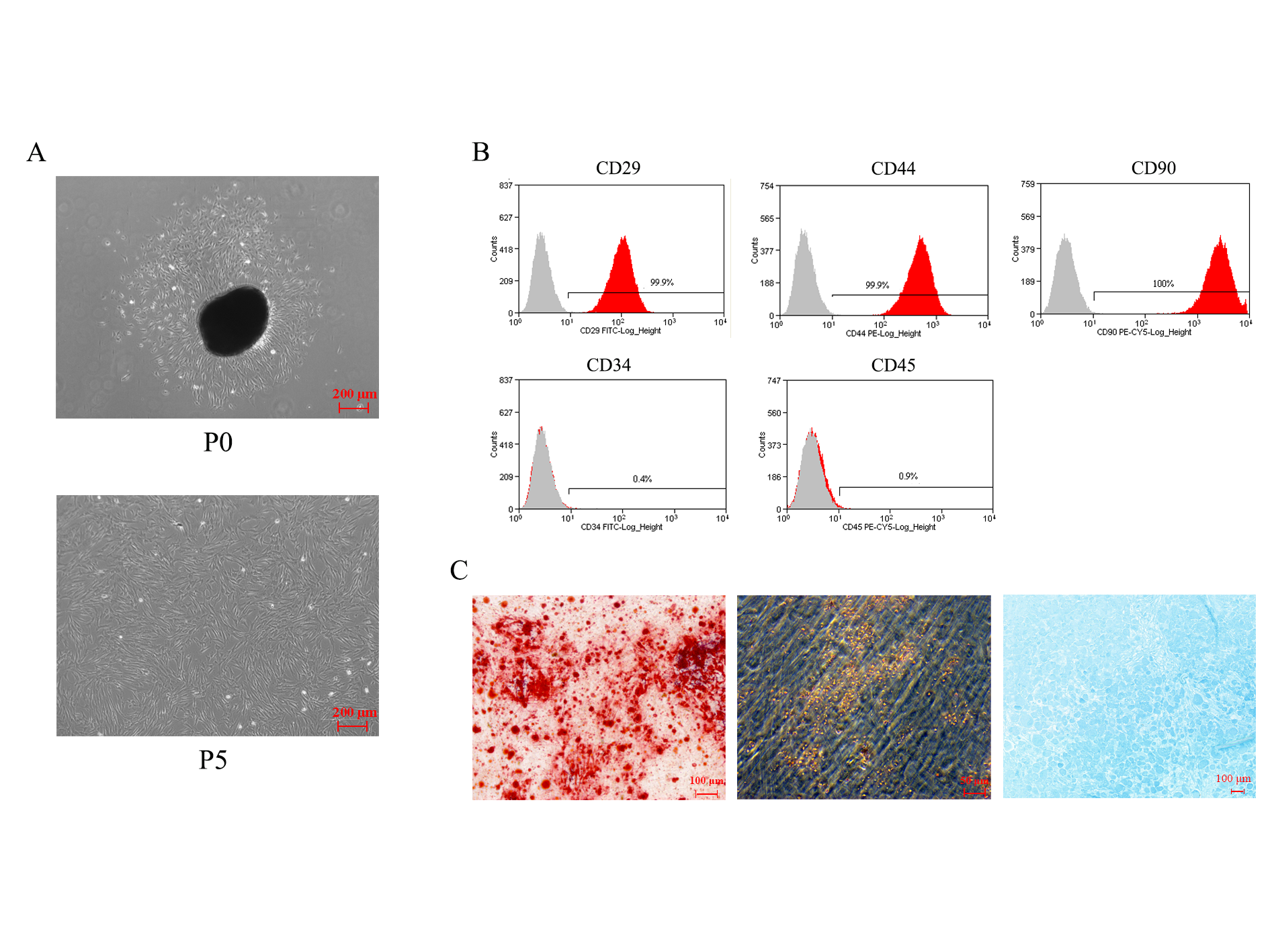

Supplement: Supplementary file 1 — Additional file 1: Fig. S1. The morphology and characterization of hDPSCs.Morphology of hDPSCs form primary culturesand the fifth passagein vitro.The expression of surface antigensin hDPSCs were detected by flow cytometry.The osteogenic, adipogenic and chondrogenic differentiation capacity of hDPSCs were verified by Alizarin red, oil red O and Alcian blue staining. [file 13287_2023_3313_MOESM1_ESM.tif]

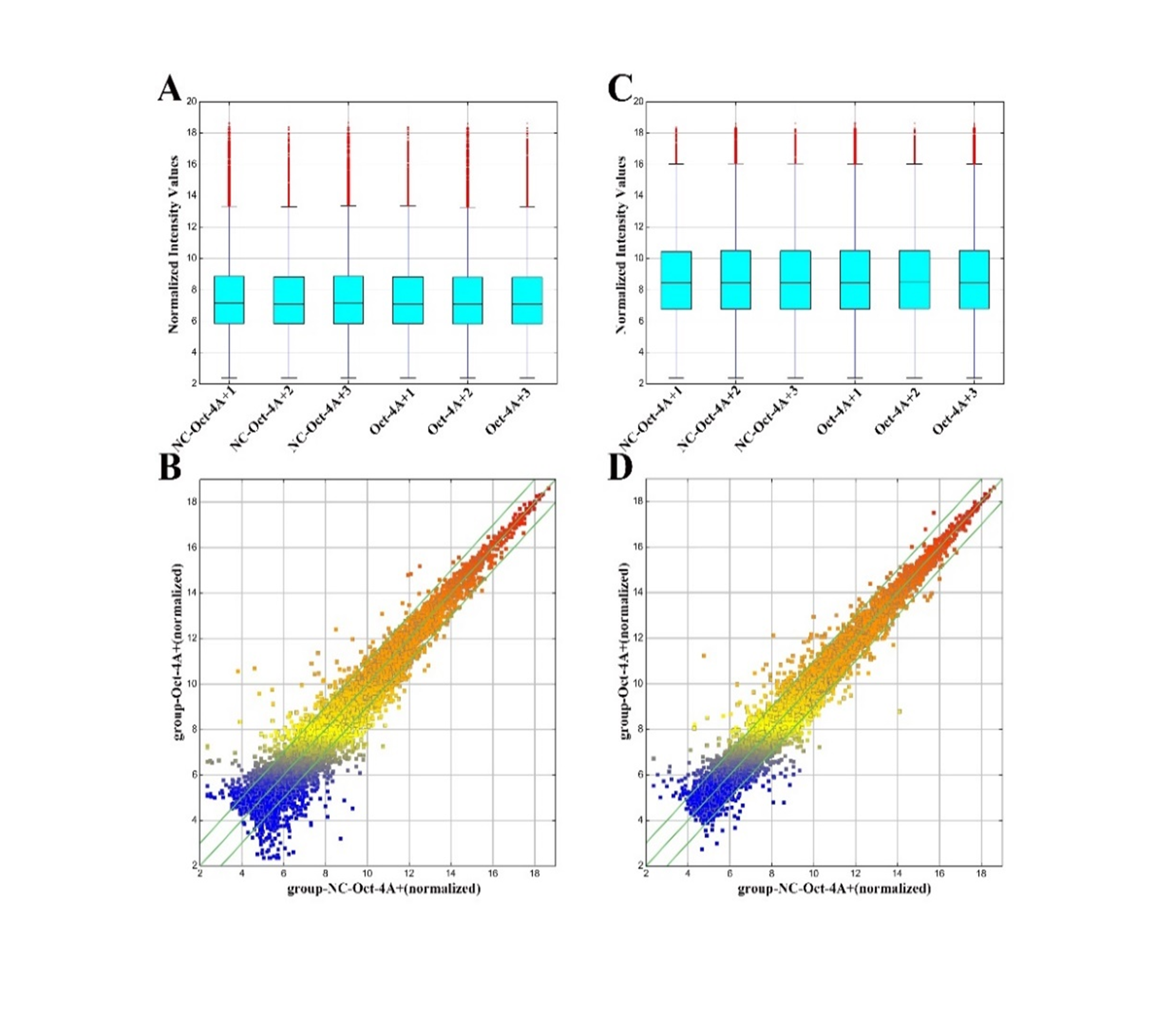

Supplement: Supplementary file 2 — Additional file 2: Fig. S2. The changes in expression profiling of lncRNAsand mRNAsin OCT4A-overexpressing and vector hDPSCs.Box plots, which permit the visualization of the dataset distributions.Scatter plots, which are convenient to visualize the variation in gene expression in OCT4A-overexpressing and vector hDPSCs. [file 13287_2023_3313_MOESM2_ESM.tif]

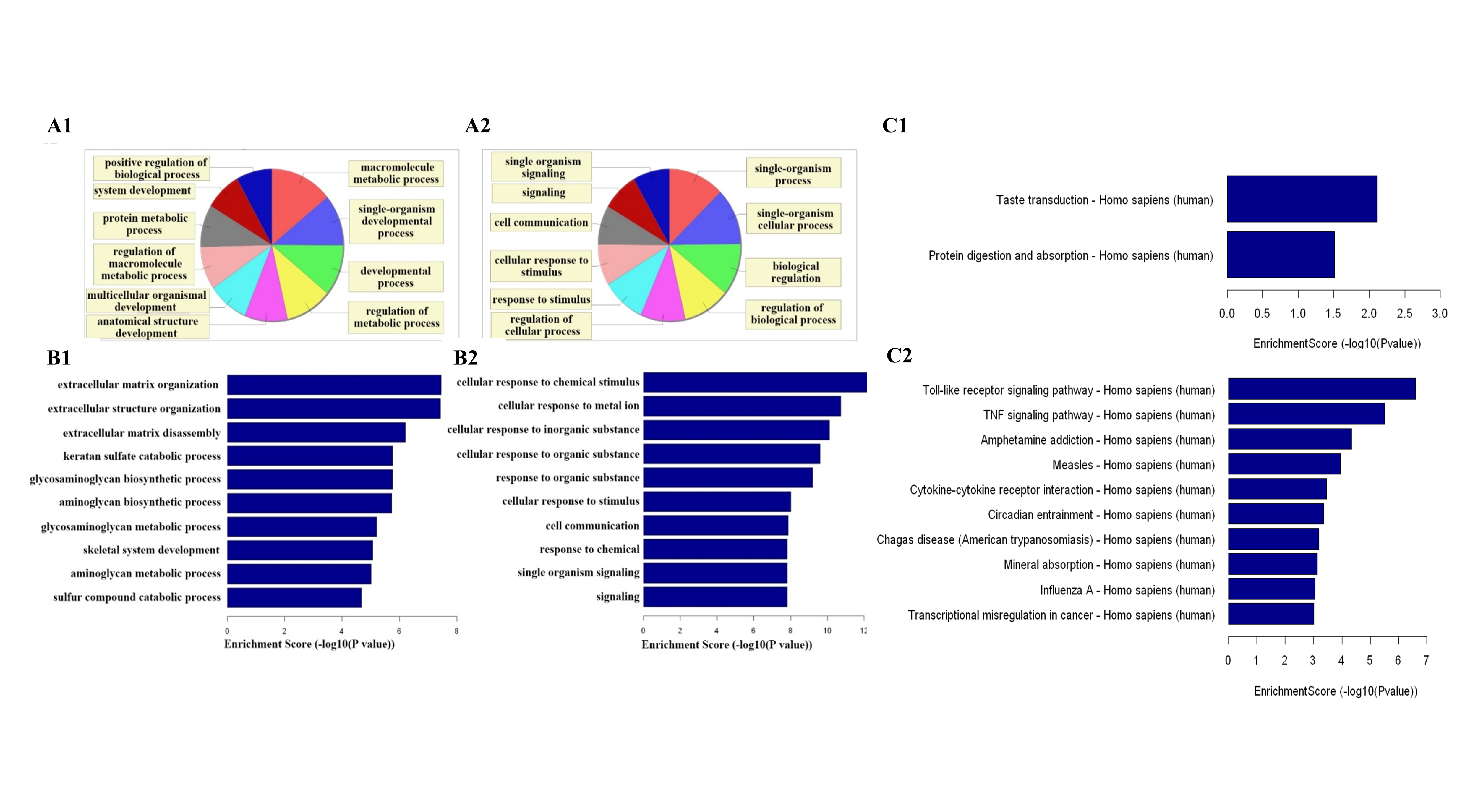

Supplement: Supplementary file 3 — Additional file 3: Fig. S3. GO and KEGG analyses of differentially expressed mRNAs in OCT4A-overexpressing and vector hDPSCs.GO analysis of differentially expressed mRNAs on biological process.Pathway analysis of differently expressed genes. GO, Gene Ontology; KEGG, Kyoto Encyclopedia of Genes and Genomes. [file 13287_2023_3313_MOESM3_ESM.tif]
