# Supplementary material for: The pluripotent factor OCT4A enhances the self-renewal of human dental pulp stem cells by targeting lncRNA FTX in an LPS-induced inflammatory microenvironment
Source: Stem Cell Res Ther. 2023 Apr 27;14:109. doi: 10.1186/s13287-023-03313-8 (PMC10142416; doi:10.1186/s13287-023-03313-8)

Fig 1E1 OCT4A (45 kDa)

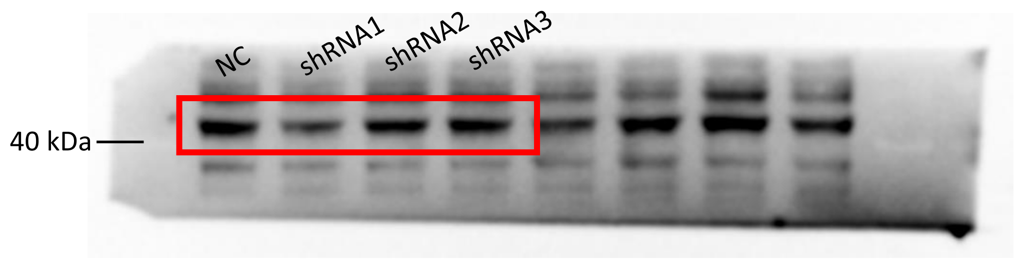

Fig 1E1 β-actin (42 kDa)

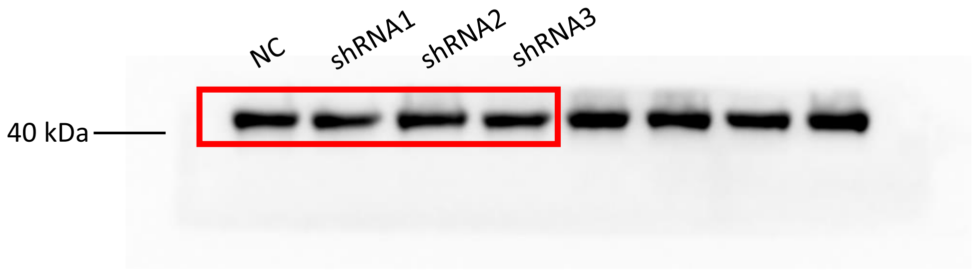

Fig. 1F1 OCT4A (45 kDa)

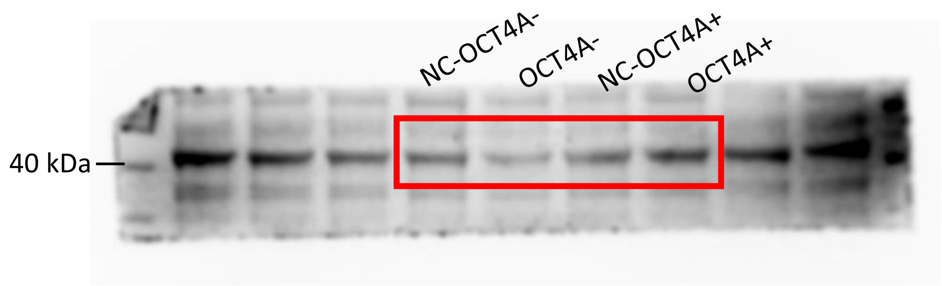

Fig. 1F1 β-actin (42 kDa)

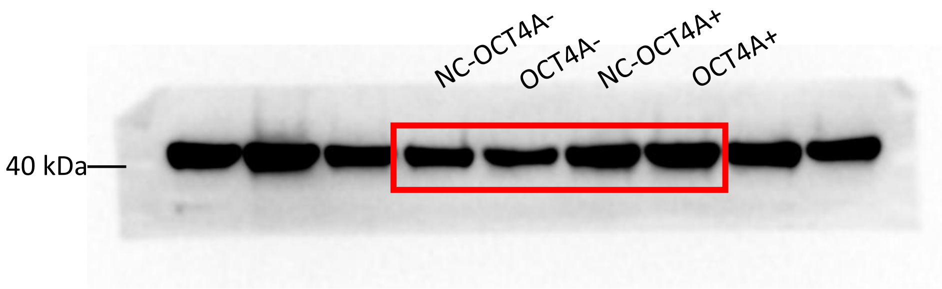

Fig. 3C1 DSPP (131 kDa)

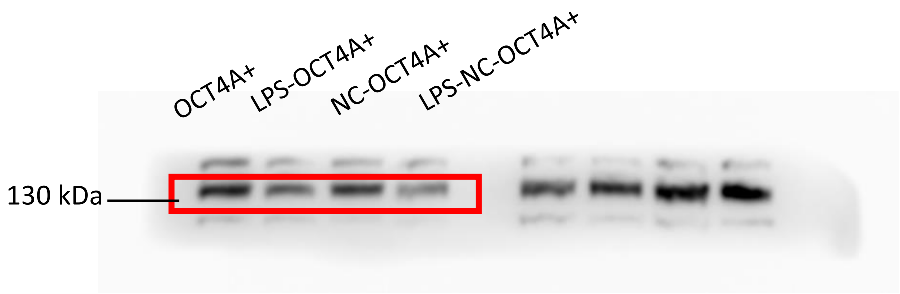

Fig. 3C1 DMP-1 (56 kDa)

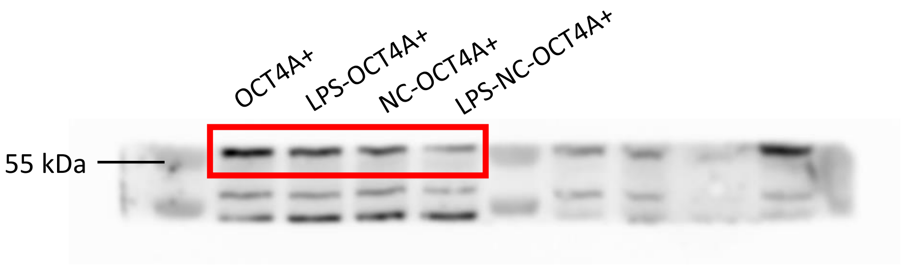

Fig. 3C1 GAPDH (37 kDa)

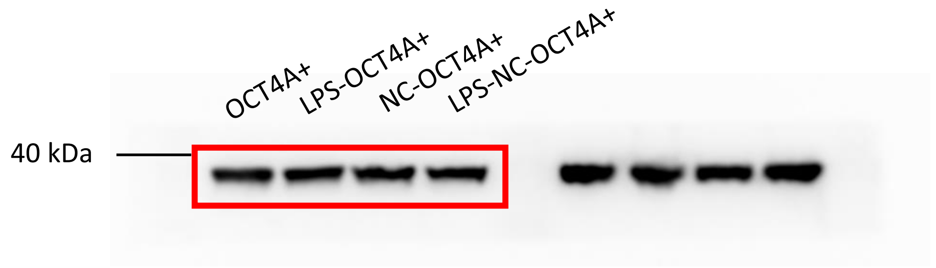

Fig. 3D1 DSPP (131 kDa)

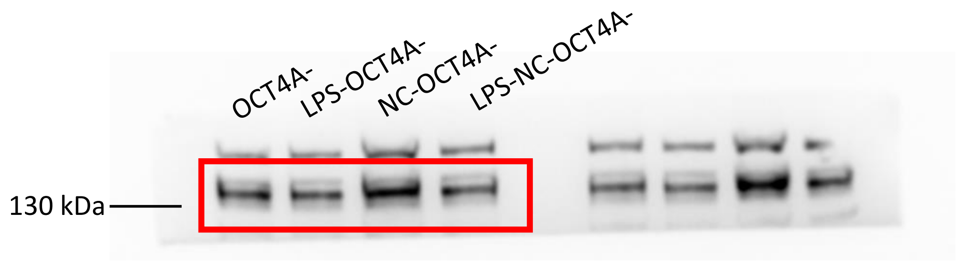

Fig. 3D1 DMP-1 (56 kDa)

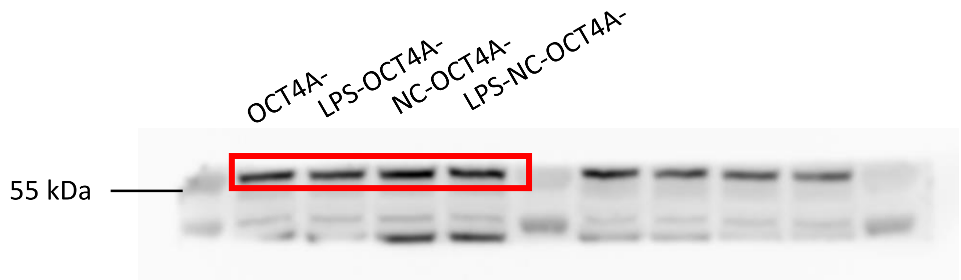

Fig. 3D1 GAPDH (37 kDa)

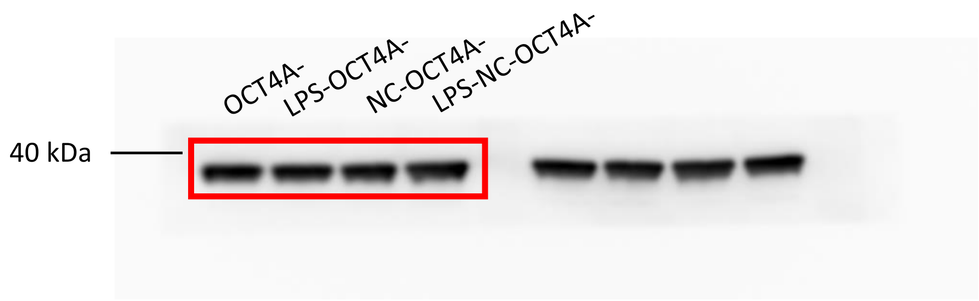

Fig. 4C1 PPARγ-2 (58 kDa)

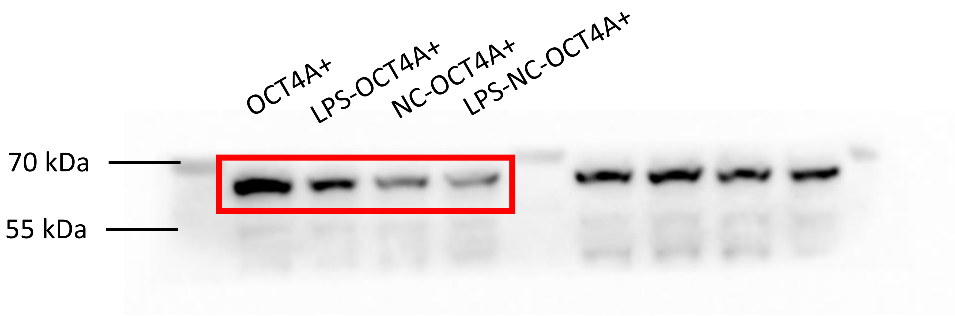

Fig. 4C1 LPL (53 kDa)

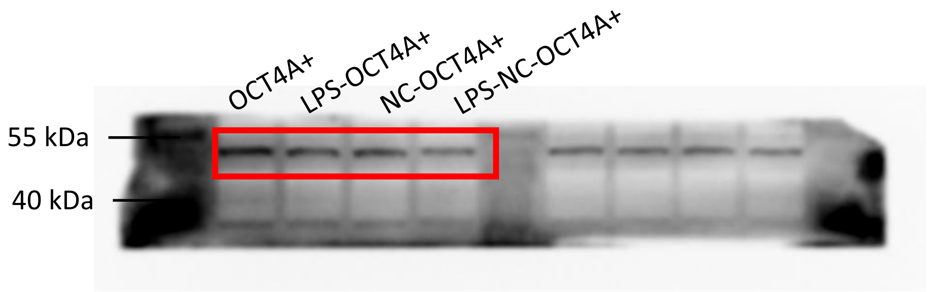

Fig. 4C1 VINCULIN (124 kDa)

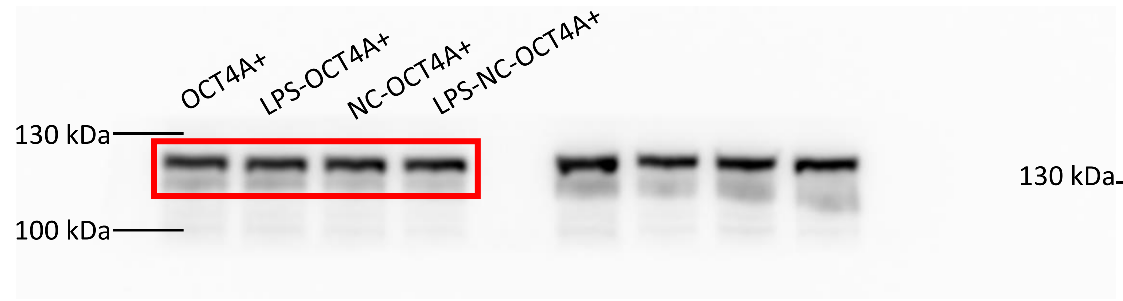

Fig. 4D1 PPARγ-2 (58 kDa)

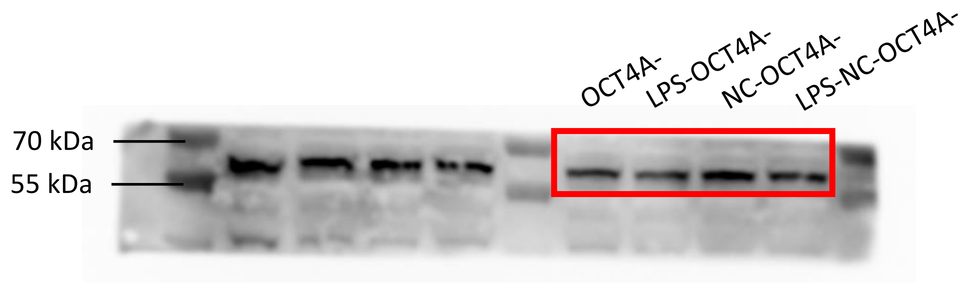

Fig. 4D1 LPL (53 kDa)

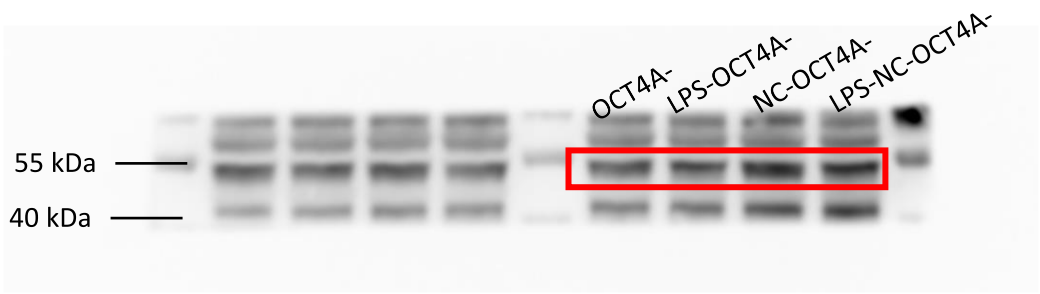

Fig. 4D1 VINCULIN(124 kDa)

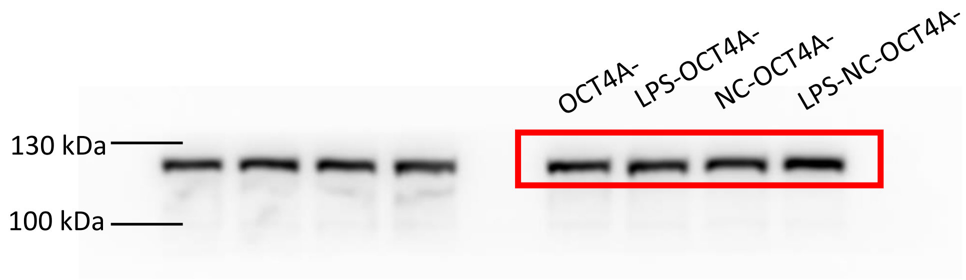

Fig. 6E1 OCT4A (45 kDa)

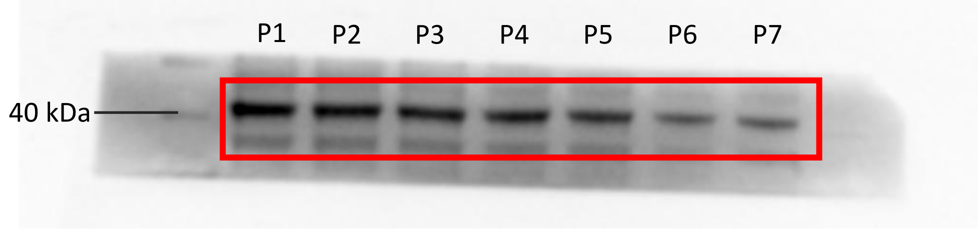

Fig. 6E1 β-actin (42 kDa)

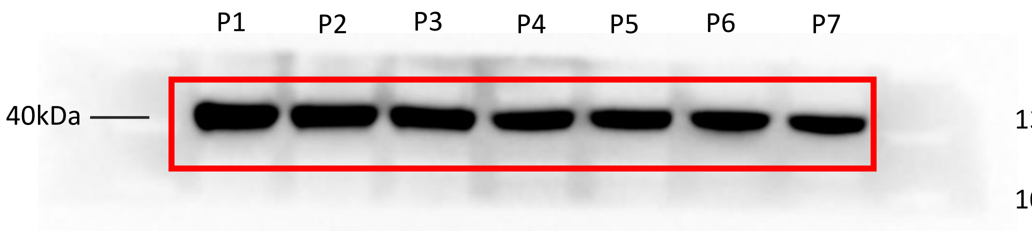

Fig. 8B1 DSPP(131 kDa)

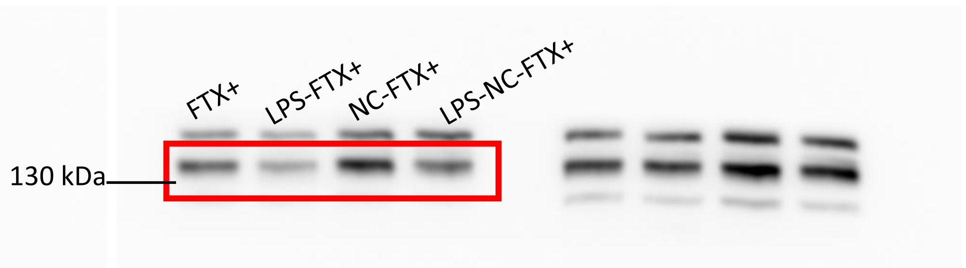

Fig. 8B1 DMP-1(56 kDa)

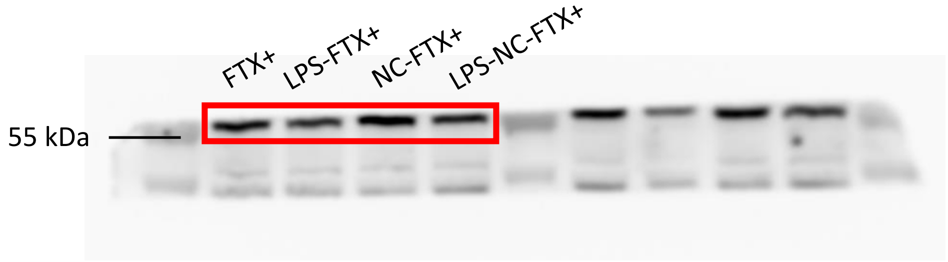

Fig. 8B1 GAPDH(37 kDa)

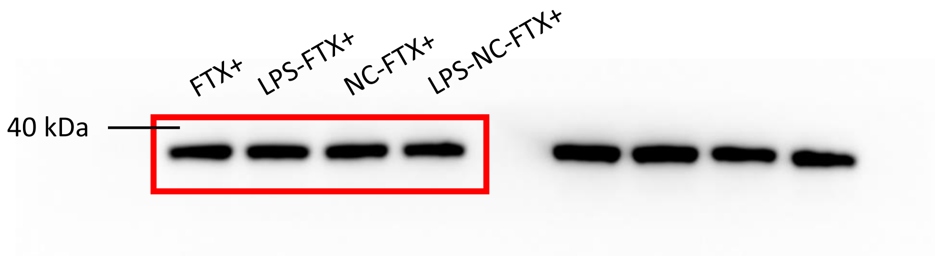

Fig. 9C1 PPARγ-2(58 kDa)

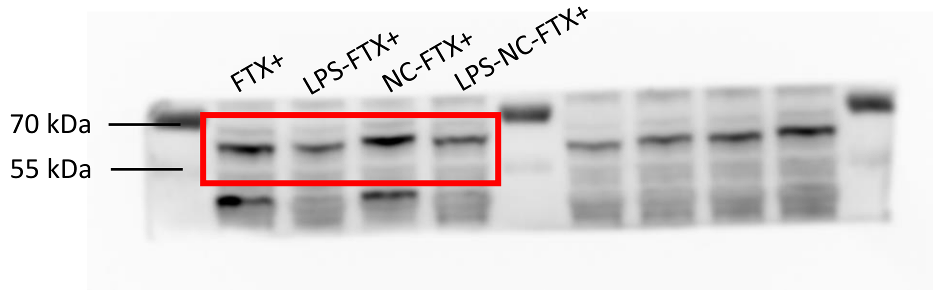

Fig. 9C1 LPL(53 kDa)

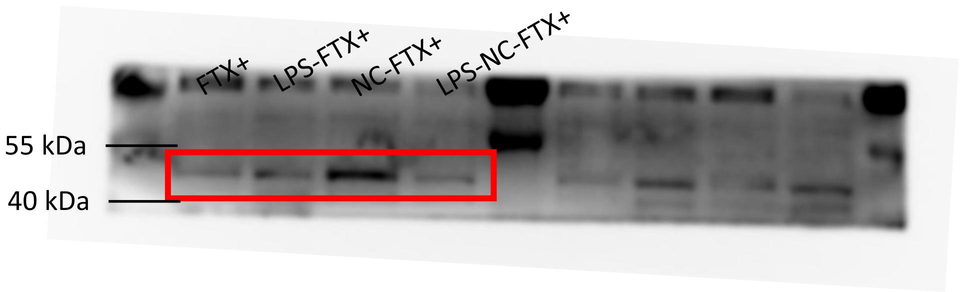

Fig. 9C1 VINCULIN(124 kDa)

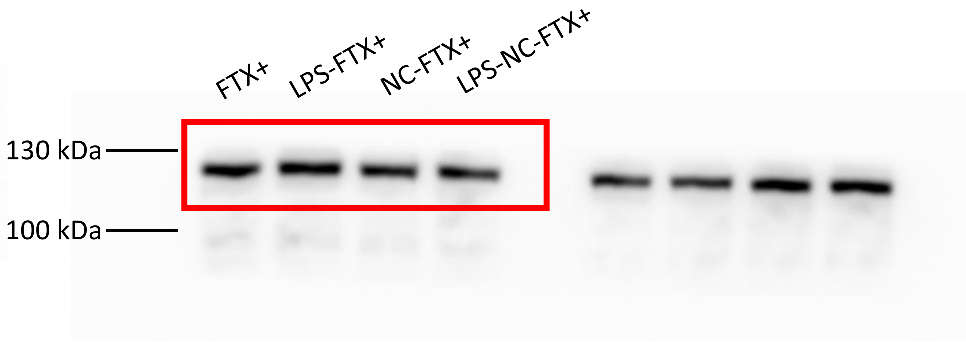

Fig. 10C

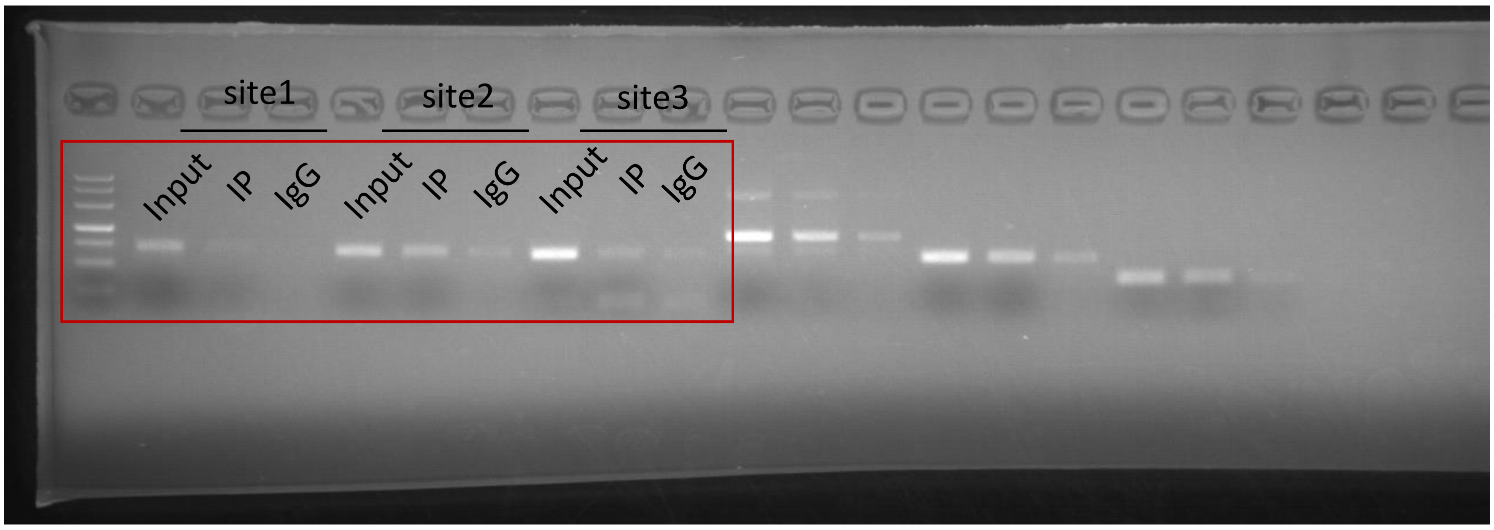

Fig 11E1 OCT4A (45 kDa)

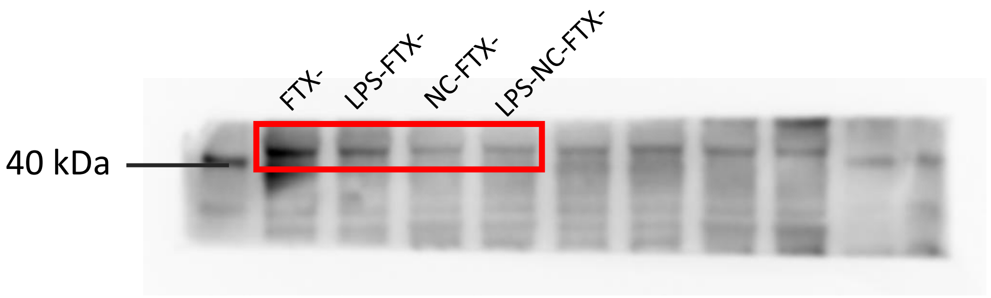

Fig 11F1 OCT4A (45 kDa)

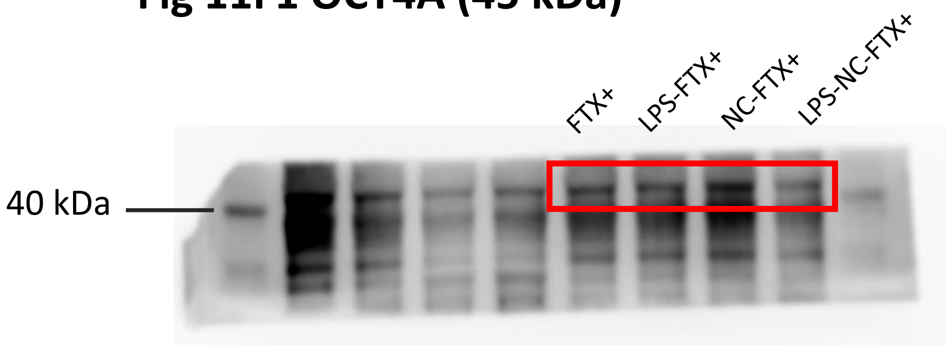

Fig 11E1 SOX2 (43 kDa)

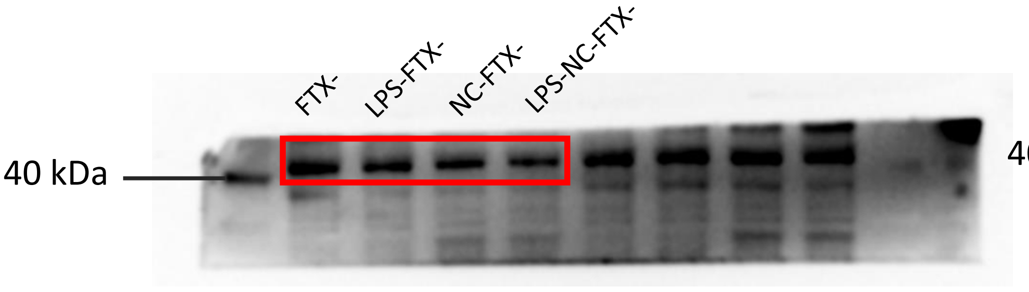

Fig 11F1 SOX2 (43 kDa)

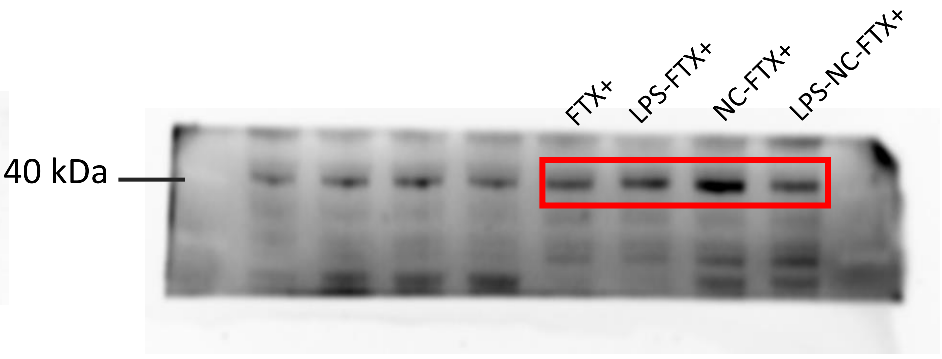

Fig 11E1 c-MYC (62 kDa)

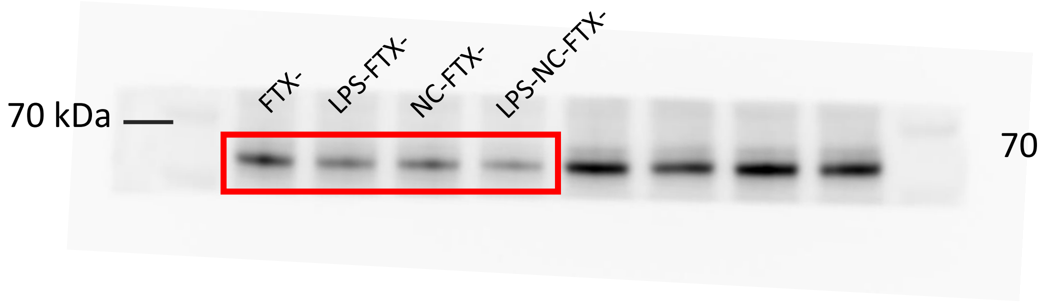

Fig 11F1 c-MYC (62 kDa)

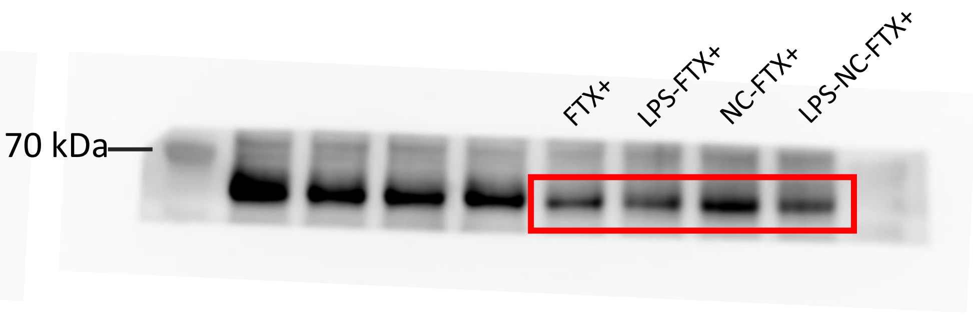

Fig 11E1 β-actin (42 kDa)

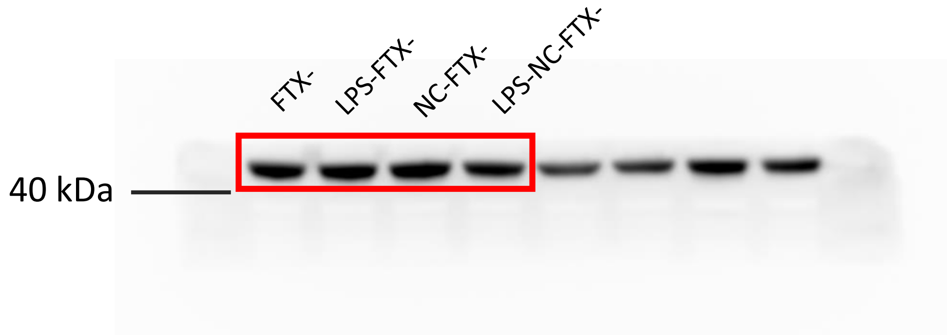

Fig 11F1 β-actin (42 kDa)

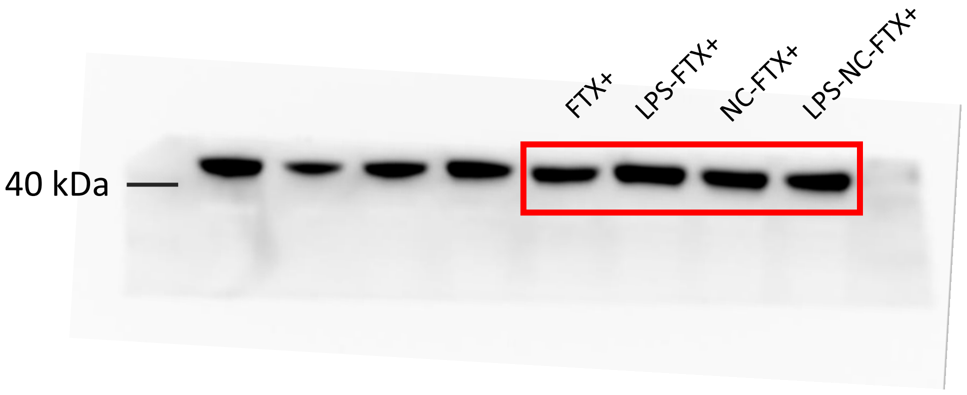

Supplement: Supplementary file 4 — Additional file 4: Uncropped gel/blot images. Uncropped gel/blot images are attached. Images used in the main figure are marked in red squares. [file 13287_2023_3313_MOESM4_ESM.pdf]
